# Misfolding, Aggregation, and Disordered Segments in c-Abl and p53 in Human Cancer

**DOI:** 10.3389/fonc.2015.00097

**Published:** 2015-04-29

**Authors:** Guilherme A. P. de Oliveira, Luciana P. Rangel, Danielly C. Costa, Jerson L. Silva

**Affiliations:** ^1^Programa de Biologia Estrutural, Instituto de Bioquímica Médica Leopoldo de Meis, Instituto Nacional de Biologia Estrutural e Bioimagem, Centro Nacional de Ressonância Magnética Nuclear Jiri Jonas, Universidade Federal do Rio de Janeiro, Rio de Janeiro, Brazil; ^2^Faculdade de Farmácia, Universidade Federal do Rio de Janeiro, Rio de Janeiro, Brazil

**Keywords:** misfolding, tumor suppressor, kinases, signaling, cancer

## Abstract

The current understanding of the molecular mechanisms that lead to cancer is not sufficient to explain the loss or gain of function in proteins related to tumorigenic processes. Among them, more than 100 oncogenes, 20–30 tumor-suppressor genes, and hundreds of genes participating in DNA repair and replication have been found to play a role in the origins of cancer over the last 25 years. The phosphorylation of serine, threonine, or tyrosine residues is a critical step in cellular growth and development and is achieved through the tight regulation of protein kinases. Phosphorylation plays a major role in eukaryotic signaling as kinase domains are found in 2% of our genes. The deregulation of kinase control mechanisms has disastrous consequences, often leading to gains of function, cell transformation, and cancer. The c-Abl kinase protein is one of the most studied targets in the fight against cancer and is a hotspot for drug development because it participates in several solid tumors and is the hallmark of chronic myelogenous leukemia. Tumor suppressors have the opposite effects. Their fundamental role in the maintenance of genomic integrity has awarded them a role as the guardians of DNA. Among the tumor suppressors, p53 is the most studied. The p53 protein has been shown to be a transcription factor that recognizes and binds to specific DNA response elements and activates gene transcription. Stress triggered by ionizing radiation or other mutagenic events leads to p53 phosphorylation and cell-cycle arrest, senescence, or programed cell death. The p53 gene is the most frequently mutated gene in cancer. Mutations in the DNA-binding domain are classified as class I or class II depending on whether substitutions occur in the DNA contact sites or in the protein core, respectively. Tumor-associated p53 mutations often lead to the loss of protein function, but recent investigations have also indicated gain-of-function mutations. The prion-like aggregation of mutant p53 is associated with loss-of-function, dominant-negative, and gain-of-function effects. In the current review, we focused on the most recent insights into the protein structure and function of the c-Abl and p53 proteins that will provide us guidance to understand the loss and gain of function of these misfolded tumor-associated proteins.

## The Folding Perspective of Misfolding

The fundamental dogma in biology for all living organisms dictates that DNA becomes RNA through the transcriptional machinery, and this step is followed by the translation of RNA to specific proteins. From the molecular point of view and using the most recent DNA technologies, it may seem easy to decode our genes to get a picture of the entire organism. However, the actual situation is a more complex and challenging scenario considering the hierarchical perspective of cells, tissues, organs, and the entire organismal network.

The next step to be elucidated in the “DNA-to-RNA-to-protein cascade” and the current challenges faced by researchers include how a linear strand of amino acids is able to minimize its free energy and conformational entropy and, ultimately, collapse into a functional architecture. To assess the reversibility of the folding/unfolding equilibrium (F–U) of a stretched amino acid sequence, several spectroscopic approaches coupled to physical perturbations are available (1–6). Depending on the sequence and length, a pure protein solution is able to shift the F–U equilibrium in a reversible manner. Uncovering this intrinsic feature of proteins to fold independently gives rise to an astonishing hypothesis: the assembly of functional protein architectures in the conformational space proceeds with precision and fidelity by itself and depends exclusively on the amino acid sequence.

From the cellular perspective, the concept of an energy landscape, in which intramolecular forces are mandatory to condense the most thermodynamically stable ensemble of conformations in a stochastic search, must now pay attention to influences from the crowded cellular milieu. The pattern of hydrophobic and polar residues in a specific amino acid sequence dictates the preferential contacts and the fingerprint for protein folding and dynamics. These key elements are likely selected and optimized during protein evolution to ensure sampling of a restricted number of conformations and ensure that proteins are “minimally frustrated” (7). The evolution of protein folding, particularly the folding of proteins containing multiple domains, provides an exquisite understanding of highly energetic substates, also known as protein intermediates. Depending on the protein architecture and secondary content, small proteins (60–100 residues) may be converted from their unfolded to their native states without populating long-lived intermediate states (two-state equilibrium). In contrast, those with more than approximately 100 residues or that are composed of at least two globular domains commonly populate intermediate states during the folding process.

Based on these assumptions, an ongoing puzzle in modern science concerns the involvement of such high-energy intermediates as driving forces to initiate pathological processes, such as several amyloidogenic disorders and cancer. During a “protein’s life” within a crowded cell, from the moment at which a nascent chain is still attached to the ribosome to the moment of death in proteasomes, amino acid chains suffer high environmental pressure to guarantee that folding takes place in a precise manner. Conversely, even considering the strictly evolutionary check-points, i.e., key amino acid interactions to avoid misfolding and a specialized endoplasmic reticulum (ER) compartment, folding is not always an infallible process.

In this review, we describe the current understanding in the field of protein misfolding and consider the formation of misfolded species, disordered segments, and aggregation and their involvement in physiological function and cancer development. We focus on the c-Abl and p53 proteins and shed light on the involvement of incorrect conformations in amyloid formation and the triggering of cell malignancy and cancer progression.

## The Cell Against Misfolding

Cell survival and proliferation are strictly dependent on multiple hierarchic pathways and a highly orchestrated network of thousands of biomolecules and cofactors. To ensure homeostasis, the building blocks of cells, i.e., protein molecules, require proper folding and dynamics to ensure their ability to work correctly and avoid cellular stress and ultimately malignant transformation. During evolution, cells acquired specialized machinery and a major housing organelle called the ER to regulate protein folding, post-translational modifications, lipid and steroid synthesis, gene expression, cellular metabolism, and calcium signaling.

The ER compartment is a “safe place” responsible for regulating the quality, folding, maturation, and trafficking of newly synthesized proteins. Features of the ER lumen are a high calcium concentration and an oxidizing environment (8) as well as a complete “army” of resident chaperones and enzymes (9, 10) to guarantee the proper folding and maturation of client proteins. The best-understood molecular chaperones are the heat shock proteins Hsp70 and Hsp90, which aid conformational maturation and target misfolded proteins for ubiquitination and proteolysis, and the chaperonins, which sequester newly synthesized proteins or misfolded ones within their structural environment for proper folding. Up to one-third of cellular proteins are synthesized within the ER (10), and most of those destined for the extracellular space are scrutinized for potential toxicity. The most common modifications in the ER factory, such as signal sequence cleavage, N-linked glycosylation, disulfite-bond formation, and glycosylphosphatidylinositol and membrane protein reshuffling and anchoring, make the process from a naive to a mature polypeptide chain slow and not always efficient. Nevertheless, secretory cells, such as hepatocytes, plasma cells, pancreatic β-islet cells, and several exocrine gland cells, manage millimolar amounts of nascent proteins at different stages of folding and assembly with extraordinary efficiency.

Interestingly, after appropriate folding and modifications, mature polypeptides are somehow sorted from misfolded species in the ER and sent to the Golgi apparatus and along the secretory pathway. Despite extended investigations to uncover the mechanism through which resident vs. client and folded vs. misfolded species are sorted in the ER for subsequent trafficking to the Golgi apparatus, this process remains obscure. Sorting models that attempt to explain this phenomenon are classified as follows: (i) receptor-mediated transport, (ii) aggregation of misfolded proteins, which restricts their ability to be transferred from the ER to the Golgi apparatus through small transport vesicles, (iii) ER retention by Golgi retrieval and (iv) the attachment of misfolded proteins to an ER matrix (11).

Dynamic control of protein synthesis, degradation, and repair dictates cell homeostasis. When the protein folding efficiency is threatened due to protein overload within the ER, cells start to experience ER stress and activate the “unfolded-protein response” (UPR). There are three well-characterized signaling sensors triggered by the UPR to overcome ER stress, i.e., inositol-requiring protein-1α (IRE-1α), activating transcription factor 6 (ATF6), and protein kinase RNA-like ER kinase (PERK). In this process, the aim is to eliminate misfolded proteins and to reduce the load of newly synthesized polypeptides within the ER. This is accomplished by decreasing the amount of mRNA available for protein synthesis, slowing the transcription/translation machinery for new mRNAs, and increasing the concentration of molecular chaperones and foldases to process accumulated proteins within the ER. Of note, the PERK branch of the UPR response was recently linked to hematopoietic stem cell (HSC) clonal integrity, in which the clearance of individual HSCs after stress prevents the propagation of these damaged progenitors (12). The remaining misfolded proteins not recovered from UPR mechanisms are sent to the ER-associated degradation (ERAD) pathways known as the ubiquitin/proteasome pathway (ERAD I) and the autophagic/lysosomal pathway (ERAD II). UPR and ERAD sensors fight to keep the cell alive and overcome cellular stress, and if these processes are unsuccessful, specialized machinery initiates programed cell death (PCD) pathways, such as apoptosis (PCD1), autophagy (PCD2), or necrosis (PCD3). A cell’s decision to live or die during cellular stress is a complex, fine-tuned mechanism that is not fully understood. For more in-depth information on UPR and ERAD mechanisms, please refer to more specialized literature (13–20).

Although evolutionary mechanisms have developed to guarantee the quality control of native protein conformations, abnormal protein synthesis is common and harmful to cells and is involved in more than 40 protein-misfolding diseases (21), including cancer, as was recently demonstrated (22, 23). The concept of a prion-like seeding mechanism is now behind the most common amyloidoses and neurodegenerative diseases (24). The tumor-suppressor p53 is a transcriptional factor that exerts broad anti-proliferative effects, including growth arrest, apoptosis, and cell senescence after cellular stress, and has been described as the most frequently mutated gene in cancer cells (25). It was recently demonstrated that subunits of the cytosolic group II chaperonin (CCT) are part of the p53 interactome (26). The correct folding of wild-type (wt) p53 requires CCT interaction, and the failure to interact with this molecular chaperone can promote the oncogenic functions of p53, even in the absence of typical DNA-binding domain (DBD) mutations (27). The correct folding of wt p53 in CCT is not a guarantee that it will safely exert its functions because mutated p53 (R248Q) aggregates into a mixture of oligomers and fibrils and sequesters the wt protein into an inactive conformation, explaining the prion-like behavior of this protein (22, 28). Breast cancer cells carrying mutated p53 exhibit a massive expression of aggregated p53 in the nucleus compared with breast cancer cells carrying wt p53, a condition that has also been shown in biopsies of breast cancer tissue (22, 29). The mechanism through which mutated p53, in association with different types of cancer, escapes the ER quality control mechanisms and triggers the dominant-negative effect of its wt counterpart is a matter of debate and is awaiting further exploration.

## The Adaptive Response of Cancer to Misfolding

The reality of cancer is that these cells exhibit genetic plasticity and adaptive advantages to survive in harmful environments. Although cancer cells adapt to trigger angiogenesis, during the growth of solid tumors, the nutrient and oxygen requirements exceed those in the surrounded vascular network. Thus, the highly proliferative and less vascularized environment of several types of cancers generates low pH (lactic acidosis), low oxygen (hypoxia), oxidative stress, and low supplies of glucose and amino acids. A small decrease in pH leads to changes in protein conformation; e.g., p53 tends to adopt a molten-globule conformation at slightly lowered pH values (30). It has also been shown that client p53 assumes a molten-globule-like state in the presence of Hsp90 (31). Depleted glucose affects protein glycosylation and ATP production, and a lack of oxygen, as an electron carrier, impairs disulfide bond formation (32). All of these factors contribute to the accumulation of misfolded proteins within the ER and the UPR. In normal cells, the stress amplitude triggers pro-survival or pro-death UPR signaling, but cancer cells escape pro-death signaling and adapt to grow under these unpleasant conditions.

The binding immunoglobulin protein (BiP), which is also known as 78-kD glucose-regulated protein (GRP78), is a chaperone and the main regulator of the UPR sensors IRE-1α, PERK, and ATF6. GRP78 inhibits the homodimerization and activity of PERK and IRE-1α (33, 34) and blocks Golgi-localization signals and further processing of ATF6 to its active conformation (35, 36). This protein was first discovered due to its upregulation in response to glucose depletion (37), a common adaptive condition known as the Warburg effect (aerobic glycolysis) (38, 39). During ER stress, increased levels of misfolded proteins bind to GRP78 in a competitive manner, leading to its dissociation from the UPR regulators IRE-1α, PERK, and ATF6. The dissociation of these UPR sensors activates ER stress signaling and culminates in the regulation of gene expression to overcome the stress condition. In tumorigenic development, downstream signaling triggered by GRP78 promotes an increase in cell proliferation, protection against apoptotic events, and the activation of tumor angiogenesis (40). Indeed, this chaperone plays a central role in the cancer adaptive response to ER stress.

An increased level of GRP78 has been reported in several solid tumors, including breast, melanoma, lung, brain, and colon (41–43), and is also associated with cancer metastasis (44). It plays a dualistic role: it can control the induction of dormancy at the beginning of tumor development but also promote pro-survival (45) and pro-metastatic functions in advanced stages (44). The localization of GRP78 to the cell surface has been shown to regulate proliferation and apoptosis in neoplastic and endothelial cells under severe ER stress (46). In prostate cancer cells, GRP78-binding partners at the cell surface, such as α_2_-macroglobulin, have been shown to increase cell proliferation (47). The use of antibodies to avoid the interaction of GRP78 with Cripto, a tumor cell-surface protein involved in the regulation of tumor progression, is sufficient to inhibit oncogenic signaling (48). In addition, ER stress has been shown to accelerate the neovascularization associated with GRP78/T–cadherin complexes (49). In contrast, the binding of GRP78 at the cell surface with Kringle-5, an angiogenesis inhibitor, is required for its anti-angiogenic and pro-apoptotic activities in stressed tumors (50). The overexpression of GRP78 protects human breast cancer cells from estrogen-starvation-induced apoptosis (51), and the binding of GRP78 with caspase-7 prevents apoptotic induction by topoisomerase inhibitors (52). Uncovering the interactome of this important chaperone associated with ER stress and the neoplastic adaptive response may provide insights for targeting cancer cells against tumorigenic development.

Heat shock protein 90 (Hsp90) is another important molecular chaperone that participates in the adaptive response of cancer cells. Together with Hsp70 and other co-chaperones, the Hsp90 complex stabilizes and activates more than 200 client proteins (53). This task is accomplished by several transient low-affinity protein–protein interactions that help Hsp90 client proteins be correctly folded or stabilized. The Hsp90 machinery is used by cancer cells to protect several mutated and overexpressed oncoproteins, such as mutated p53 and Bcr–Abl, from misfolding and degradation. However, it is also involved in normal cellular physiology, including nuclear processes such as those involved in transcription, chromatin remodeling, and DNA damage-induced mutation (54).

The transcriptional repressor BCL-6 regulates ataxia telangiectasia and Rad3-related (ATR) and TP53 gene expression (55). The Hsp90 and BCL-6 protein complex represses ATR and TP53 expression in diffuse large B cell lymphomas. Hsp90 inhibition has been shown to decrease BCL-6 levels due to protein instability, leading to the activation of target genes (ATR and TP53) and the apoptosis of lymphoma cells, showing that the Hsp90–BCL-6 interaction is crucial for lymphoma survival (56). Wt p53 is a short-lived protein that turns over through the ERAD I machinery (57). Hsp90 has been shown to bind wt p53 (31, 58) and is necessary for protein stability and proper DNA-binding ability at physiological temperatures (59, 60). However, Hsp70 is also required to support p53 activity under stress (61). Surprisingly, Mdm2, a negative regulator of p53, has been shown to chaperone against p53 (62). When mutated, however, p53 has an increased intracellular half-life due to impaired ERAD I degradation processing (63). Part of this impaired mechanism occurs because of the aberrant physical association of mutated p53 with the Hsp70 and Hsp90 molecular chaperones, which may protect the protein from ERAD I processing (64, 65). Geldanamycin (GA), a selective Hsp90 inhibitor, is able to restore the ERAD I processing of mutated p53 in tumor cells but is not effective at restoring its transcriptional factor function (63, 65).

Geldanamycin and other benzoquinone ansamycins not only target mutated p53 through Hsp90 inhibition but are also effective at dissociating several kinase–Hsp90 complexes and thereby alleviating downstream signaling pathways and kinase-induced oncogenic transformation (66–68). In chronic myeloid leukemia (CML) or acute lymphoblastic leukemia (ALL), a reciprocal translocation between the *bcr* and *c-abl* genes produces the unregulated kinases p210^Bcr–Abl^ and p185^Bcr–Abl^ (69). In this type of cancer, GA has been shown to sensitize Bcr–Abl-positive cells to cytotoxic chemotherapy (70). Several other drugs, including novel oxime derivatives of radicicol (71) and novobiocin (72), have been shown to be effective for the therapeutic intervention of CML by disrupting the Bcr–Abl–Hsp90 complex. Because Bcr–Abl is destabilized and degraded upon Hsp90 inhibition, it may represent a new opportunity for blocking CML progression in Bcr–Abl mutations associated with a drug-resistant phenotype (73). T315I p210^Bcr–Abl^, the most aggressive and insensitive mutation to the first and second generation of tyrosine kinase inhibitors, has been shown to remain sensitive to Hsp90 inhibition and to suppress leukemic stem cells in a mouse model (74). Although Hsp90 participates in the protein stability of several oncogenic kinases, the molecular mechanisms underlying these interactions have not been fully elucidated. Recently, a kinase inhibition study contributed to uncovering the conformational plasticity of kinases during Hsp90 interaction. For Bcr–Abl, the disruption of the kinase–Hsp90 complex has been shown to be independent of whether the chimeric protein was in an active or inactive conformation, but this was not true for other kinases (75).

**Figure 1:**
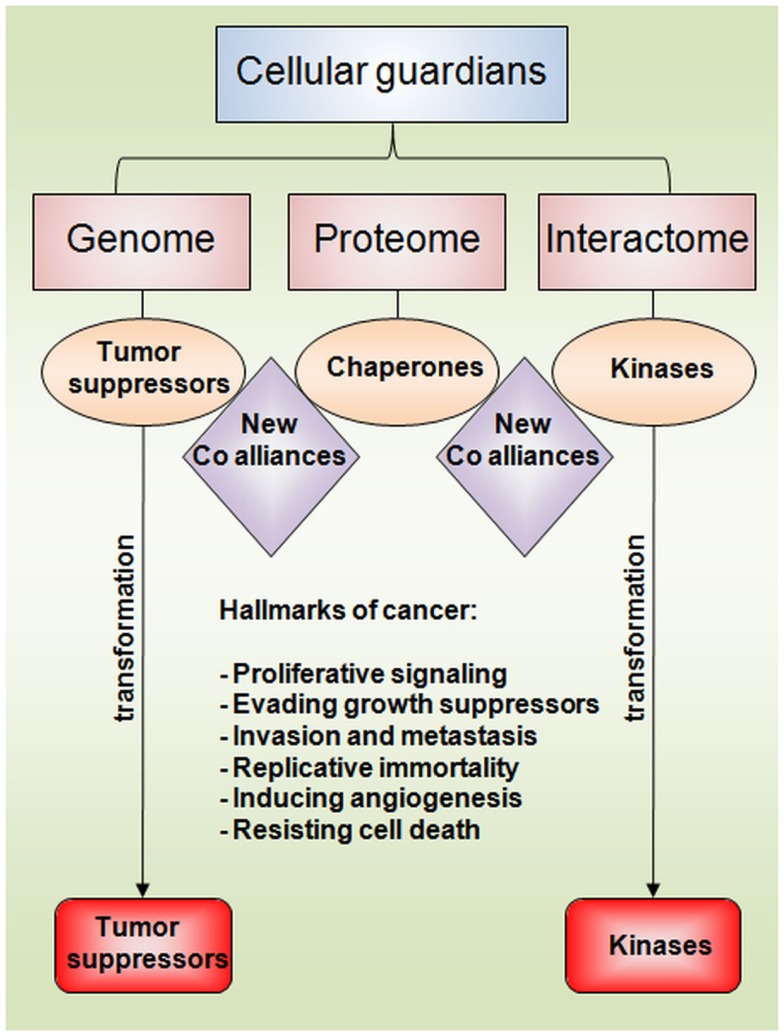
**Schematic representation of cellular guardians**. Tumor suppressors, chaperones, and kinases represent genome, proteome, and interactome examples of cellular guardians, respectively. The maintenance of tumorigenic processes is commonly achieved via new co-alliances and the transformation of different guardians. The hallmarks of cancer were highlighted in the scheme.

Using an analogy from the medieval era in which guardians from different clans worked together to defend the empires of their kings, cellular homeostasis and survival are maintained under the control of genomic, proteomic, and interactomic guardians (Figure 1). The opportunist behavior of cancer cells to make new guardian co-alliances and to transform and manipulate them to their own benefit may provide an explanation for the maintenance and progression of these neoplastic diseases and their hallmarks (76).

## The Impact of Unfolded p53 Segments on Its Functions and Cancer Development

Tumor suppressors are complex macromolecules normally occurring as multi-domain proteins flanked by disordered segments. The impact of this architecture on protein activity and cellular function is beyond our current understanding, even using the most recent state-of-the-art methods in structural biology. Three well-organized domains have been recognized in p53: an N-terminal transactivation domain (TAD, spanning residues 1–70), a sequence-specific DBD (residues 94–293), and an oligomerization domain (OD, residues 324–355). Flanking these regions, there are two disordered polyproline regions (PPRs): the first is composed of residues 71–93, which link the TAD to the DBD, and a second spans residues 294–323 and links the DBD to the OD. At the most extreme C-terminal region (residues 356–393), an unstructured basic region ends p53.

The TAD region does not fold independently (77, 78) but acquires a pair of helices upon binding to the nuclear coactivator binding domain of the CREB-binding protein (CBP) (79, 80). The p53 activity and stability are regulated depending on the TAD phosphorylation of specific serine and threonine residues. The transcriptional factors p300/CBP and the ubiquitin protein ligase Mdm2 (Hdm2 for the human ortholog) have overlapping binding sites within this N-terminal region. Upon DNA damage, phosphorylation at Ser15, Thr18, and Ser20 results in the dissociation of Mdm2 from TAD and an increase in p300/CTB affinity, thus facilitating p53 transcriptional activity (81, 82). Conflicting data exist concerning the participation of phosphorylated Ser15 in regulating the ability of p53 to complex with Mdm2 or to mediate p53 degradation (83–85). A direct effect of Mdm2 binding to TAD is the inhibition of p53 transcriptional function. However, the major effect on p53 occurs through its E3-ubiquitin ligase activity (86). The p53–Mdm2 interaction has been shown to be critical when the lethality of *mdm2*-null mice was rescued by simultaneous deletion of the TP53 gene (87, 88). The nuclear export signaling (NES) of p53 is triggered by the mono-ubiquitination of several lysine residues, which results in a blockade of its transcriptional activity. Otherwise, poly-ubiquitination occurs, which acts as a signal for degradation in the nucleus or cytoplasm (89). In addition to binding to the TAD, a second Mdm2-binding site was identified in the p53 core domain and has been speculated to stabilize the Mdm2–p53 interaction during degradation (90). The incorporation of specific modifications to p53, such as NEDDylation, is also dependent on Mdm2 expression levels and the direct binding of Mdm2 to p53. The Mdm2 RING finger E3-ubiquitin ligase has been shown to be NEDDylated, and after this step, NEDD8 can be conjugated to the C-terminus of p53 to inhibit its activity (91). The status of ubiquitin binding to p53 is also determined by Mdm2 protein levels. The p53–Mdm2 binding is quite enigmatic and works through negative feedback. The activation of p53 induces the transcription of Mdm2, and the accumulation of Mdm2 inhibits p53 activity. Additionally, Mdm2 is able to block its own transcription (92).

Because a complete loss of transcriptional activity has only been observed with additional substitutions targeting residues Trp53 and Phe54 (93), it has been suggested that the p53 TAD has multiple binding elements, which are able to interact with transcription factors and other regulators. The presence of multiple post-translational modifications and the flexibility of this region were likely selected during protein evolution to serve as multiple binding sites and consequently provide a fine-tuned mechanism for the regulation of p53 activity and stability (94). The exposure of normal cells to genotoxic agents or non-genotoxic stresses leads to p53 phosphorylation at approximately 15 serines or threonines in both the N and C-termini and to the acetylation of approximately six lysines in the C-terminus (95, 96). Therefore, the end regions of p53 act as molecular antennas for the proper activity and interactome signaling of this tumor suppressor.

MdmX (HdmX for the human ortholog) also participates in p53 signaling and inhibits p53 transcriptional activity (97–99). MdmX and Mdm2 may work as partners (100, 101) and directly contribute to tumor formation, as observed by the immortalization and neoplastic transformation of retrovirus-mediated MdmX overexpression in primary mouse embryonic fibroblasts (102). Several tumor cell lines express increased levels of HdmX compared with normal cells (103). The systematic screening of HdmX expression in more than 500 human tumors of different origins has revealed HdmX overexpression in a wide range of these tumors (102), suggesting that HdmX may function as an oncogene. Moreover, MdmX has been shown to block the p300/CBP-mediated acetylation of p53 (104), a modification involved in the tumor-suppressor functions of p53 (105).

The first disordered PPR of p53, which spans residues 71–93, was demonstrated to bear five partially conserved PxxP motifs and to participate in protein activity and regulation. The depletion of this PPR does not influence p53 transcriptional transactivation but severely affects growth suppression (106). This finding shows that p53 transcriptional activity and growth suppression are uncoupled events and that the first PPR region mediates a critical activity in p53-dependent tumor suppression. Further studies have identified this region as crucial for p53-mediated apoptosis but dispensable for cell growth arrest and the suppression of cell transformation (107). The absence of the first PPR in p53 has been shown to decrease both the specificity of target promoters and the induction of apoptotic genes, such as *pig3*, *pig6*, *pig11*, *p85*, and *btg2* (108). This region is also important for p53 regulation because the lack of PPR increases the Mdm2 affinity for p53 and makes it more susceptible to the negative regulation of Mdm2, facilitating protein ubiquitination and nuclear export (109). Further explorations have clarified the mechanism through which PPR renders p53 sensitivity to Mdm2 inhibition. The Pro82 located in the first PPR is required for p53/Chk2 interaction in response to DNA damage and subsequent Ser20 phosphorylation (110). Germline substitutions (e.g., P82L) and somatic mutations (e.g., P85S and P89S) in bladder tumors (111) in subjects with Li–Fraumeni syndrome and ovarian carcinoma (112) have shown that the first PPR of p53 plays an important role in regulating protein activity. Although classified as a transcription factor, p53 can also mediate apoptosis without new RNA and protein synthesis. This transcription-independent mechanism has been observed in human vascular smooth muscle cells in which p53 activation is able to increase surface Fas (CD95) expression by transport from the Golgi complex (113).

The second PPR located between the DBD and OD contains seven proline residues. As indicated by the presence of several prolines within the first and second PPRs, a cautionary note about these segments concerns the effect of the number of prolines on the kinetics of p53 folding. Proline regions have conformationally constrained backbones that may not only interfere with proper p53 folding but also allow multiple symmetrical orientations among the TAD, DBD, and OD to enhance the induced fit conformations upon protein or DNA binding.

The last disordered segment of p53 concerns the basic C-terminal region (BR) that is also prone to multiple post-translational modifications. The presence of multiple acetylated lysines within the p53 BR makes it similar to histone tails, not only physically but also functionally. The binding of p300/CBP acetyltransferase to p53 TAD is able to acetylate not only histones but also p53 itself (114). A series of studies have been conducted in an attempt to uncover the complex role and regulation of p53 acetylation. Two groundbreaking studies have shown conflicting results concerning the involvement of p53 acetylation in triggering its DNA-binding function. First, Gu and Roeder concluded that p53 acetylation by p300 occurs in the C-terminal domain and is critical for stimulating the DNA-binding function of p53, likely due to an acetylation-induced conformational change (114). Later, Espinosa and Emerson showed that the acetylation of the C-terminus by p300 is not necessary for p53 DNA binding or promoter activation (115). Further exploration revealed the key steps in the mechanism of p300–p53–DNA transactivation. Phosphorylation by Chk2 at Thr18 and Ser20 in the p53 TAD stabilizes p300 docking to the TAD domain (116). This docking was shown to be essential for the DNA-dependent acetylation of p53, suggesting that the acetylation sites within the p53 tetramer are occluded in the absence of DNA (117). Furthermore, the role of acetylation as a post-DNA-binding process is important for clamping and stabilizing the p300–p53 acetylated complex (117). Finally, to identify the p300-docking motifs in p53 related to protein acetylation, p300 was subjected to peptide selection from a phage-peptide library. The identification of a second flexible p300-binding motif within the PPR of p53 has been shown to be required for acetylation and p53 binding to promoter sites (118). These observations provide a mechanism to explain how p300/CBP binding to p53 increases protein stability and transcriptional activity. The p53 BR has been shown to be acetylated by p300/CBP at Lys372, 373, 381, and 382 (119–121). P/CAF, a histone acetyltransferase (HAT) associated with p300/CBP, has also been shown to acetylate Lys320 within the second PPR (119). Acetylation at position 373 in p53 by p300/CBP leads to cell apoptosis, whereas acetylation at 320 by P/CAF leads to cell-cycle arrest (122). In colorectal cancer (CRC), ArhGAP30, a Rho GTPase-activating protein, is a crucial regulator of p53 acetylation and activity. This protein binds to the C-terminus of p53 and facilitates the p300-mediated acetylation of p53 at Lys382. A low expression of ArhGAP30 is correlated with poor survival in CRC patients, showing that ArhGAP30 is a potential marker of CRC (123). This finding shows how p53 acetylation works in a fine-tuned mechanism that affects the gene-expression patterns and cell fate in normal physiology and cancer development.

The BR of p53 binds to non-specific sequences in DNA (124–126) and also regulates the sequence-specific binding of the core (127, 128). Using analytical ultracentrifugation, an inverse relationship was observed between the number of acetyl groups attached to the C-terminus of p53 and its ability to bind DNA (129). More recently, electron microscopy and single-molecule experiments have provided a basis to understanding how full-length p53 scans and recognizes specific DNA-responsive elements. The synergistic model of scanning and DNA recognition is based on the ability of the C-terminus to rapidly translocate as the core domain hops along DNA with transient associations for rapid scanning (130–132).

The transfer of acetyl groups to the ε-amino group of lysine residues in histones represents one of the best characterized post-translational modifications for chromatin regulation. Acetyl groups are also transferred to non-histone proteins, as shown above for p53, and modulate protein function by changing stability, cellular localization, and protein–nucleotide/protein–protein interactions. The acetylation status is maintained by the opposing activities of HATs and histone deacetylases (HDACs) in a controlled manner. In the case of p53, HDAC-1, -2, and -3 are all capable of downregulating p53 function, showing that the deacetylation of p53 is part of the mechanism that controls the physiological activity of p53 (133). However, AMP-activated protein kinase (AMPK) phosphorylates and inhibits the p53 deacetylase SIRT1, promoting p53 acetylation and apoptosis in hepatocellular carcinoma (134). This finding shows that HDACs are also involved in cancer development. In CML cells with Bcr–Abl-independent imatinib resistance, the resistance mechanism includes the aberrant acetylation of p53 and other proteins due to the upregulation of HDACs and the downregulation of HATs, indicating HDACs as targets for imatinib-resistant leukemia cells (135). In contrast, the pathogenic protein AML1/ETO that is involved in t(8;21) acute myeloid leukemia has been shown to be proteasome-degraded when treated with panobinostat, a HDAC inhibitor, and does not require functional p53 or the activation of conventional apoptotic signaling in a mouse model (136).

## The Effect of p53 Mutations and Aggregation on Its Functions and Cancer Development

The evaluation of 3,281 samples of 12 different tumor types revealed 127 mutated genes in different signaling and enzymatic processes, and the TP53 gene was detected as the most frequently mutated gene (42% of samples) (25). The current database of TP53 mutants (http://p53.fr) reveals 45,000 somatic mutations, most of which provide advantages to a specific cell clone in its microenvironment, increasing its survival or reproduction. These driver mutations normally trigger clonal expansions and tumorigenic development (137).

Factors influencing the TP53 mutational frequency in tumors may be classified by their high heterogeneity and the different cancer subtypes, the stage of cancer development, and ambient factors, such as viral and bacterial infection. Depending on the cancer type, TP53 mutations can range from <5%, as in cervical carcinoma, to 90% in ovarian carcinoma. For instance, a molecular search in breast carcinoma revealed four major subtypes of TP53 mutations with variable frequency, ranging from 12% in luminal A and 30% in luminal B to 72% in HER2-E and 80% in basal-like (138, 139). Considering the stage of development, a low frequency of TP53 mutations was reported for primary prostate tumors (between 10 and 20%) in contrast to metastatic tumors (up to 50%) (140). In CML disease, the occurrence of TP53 mutation is more frequent in the blastic phase (141, 142). Concerning exogenous factors, most human viruses impair p53 activity. In cervical cancer, the human papillomavirus E6 protein targets p53 for degradation (143). Bacterial infection has recently been shown to trigger the p53 pathway and to activate p53 isoforms (144). Moreover, the p53 R249S variant is often observed in liver cancer as being associated with aflatoxin B1 food contamination (145).

Mutations affecting amino acid sequences (i.e., missense mutations) are commonly observed in the TP53 gene. In the case of TP53, monoallelic alterations occur within six hot-spot sites in the DBD (R175, G245, R248, R249, R273, and R282). Even though missense mutations are predominant in tumors, other genetic alterations have already been described in TP53 (146), as observed in osteosarcoma with a high frequency of TP53 gene deletion (147, 148). Furthermore, TP53 germline mutations cause a rare autosomal-dominant cancer predisposition called Li–Fraumeni syndrome (149, 150). Most predisposed subjects present a variety of tumor types and carry a specific p53 germline mutation with approximately 90–95% penetrance (151). Similarly, dominant-negative mutations in TP53 are involved in tumor growth and development in glioblastoma (152). It is clear that we are still far from fully comprehending the complex behavior of TP53 mutations and signatures in human cancer and its effects on the p53 protein network, even considering that TP53 expresses eight differentially spliced mRNAs and is translated into 12 isoforms.

In fact, there is no unanimous resolution for the following question: in what circumstances can p53 act as a tumor suppressor or oncogene? The transcriptional activity of p53 may range from total inactivation to an activity greater than that of the wt form (153). The high frequency of p53 mutations in several tumors and the fact that p53^−/−^ mice display marked early onset and cancer predisposition depict p53 as an important tumor suppressor (94, 154). In contrast, mouse models have shown that to work as a tumor suppressor, p53 does not respond to acute DNA damage but to the oncogene-induced expression of the p19^ARF^ tumor suppressor, which activates p53 via the sequestration and inhibition of Mdm2 (155, 156). In addition, mice deficient for p21, Puma, and Noxa are not able to trigger p53-mediated apoptosis, G1/S cell-cycle arrest, and senescence but remain free of tumor development for at least 500 days compared with p53-null mice. This finding suggests that the induction of apoptosis, cell-cycle arrest, and senescence is dispensable for the p53-mediated suppression of tumor development and that genomic stability or metabolic adaptation are more important for p53 suppressor activity (157). Surprisingly, mice bearing three lysine-to-arginine substitutions at p53 acetylation sites (K117R, K161R, and K162R) retain the ability to regulate energy metabolism and reactive oxygen species production compared with p53-null mice, showing that metabolism regulation and antioxidant function are crucial events for the suppression of early onset tumorigenesis (158). Knock-in mice expressing an allelic series of p53 TAD mutations have revealed that the transactivation of p53 is essential for tumor suppressor activity but is associated with a small set of novel p53 target genes (159).

p53 mutations may lead to different effects: (i) mutant p53 may lack the activity of wt p53 (loss-of-function – LoF) (ii) mutant p53 may acquire oncogenic activity without disturbing wt p53, (iii) mutant p53 may inhibit wt p53 through a dominant-negative effect and trigger oncogenic functions and (iv) mutant p53 may inhibit wt p53 protein through a dominant-negative effect and reduce activity. The acquisition of oncogenic activity by mutated p53 was first revealed in experiments using the transfection of mutant p53 into TP53-null cells, which revealed the formation of tumors in mice (160–162). In addition to the gain of function, mutated p53 may operate through a dominant-negative mechanism, with the formation of hetero-oligomers between the mutant protein and wt p53 (163, 164). A possible dominant-negative effect may also be observed with mutated p53 and its ancestral p63 and p73 paralogs. In contrast, mass spectrometry experiments have revealed that p63 and p73 homotetramers are able to form mixed tetramers after 30 min of incubation. Conversely, neither p53 and p73 nor p53 and p63 homotetramers were able to exchange components after a 24-h incubation, showing a divergent evolution of the oligomerization domain within the p53 family (165). Furthermore, a gain-of-function phenotype of mutated p53 has been shown by the co-aggregation of p63 and p73 (166), showing that the creation of dominant-negative p53 through hetero-oligomerization is not exclusive. It is likely that dominant-negative mechanisms occur via high-level oligomeric states, in which aggregated mutant p53 sequesters wt p53 into mixed oligomers (22, 23). Several oncogenic functions of mutated p53 have been characterized, and for more in-depth information, please refer to specialized literature (167, 168). Of note, the c-Abl kinase also forms homo- and hetero-oligomers with its binding partner Abi-1 in a kinase-dependent manner (169). In the chimeric Bcr–Abl protein, the oligomerization domain of Bcr has been revealed to be crucial for the transforming function of this aberrant protein (170), but no relationship has been demonstrated in terms of the involvement of higher oligomeric states in leukemogenesis. Few works have linked protein aggregation to cancer development. Another important tumor suppressor that is inactivated in several types of cancer, the retinoblastoma (Rb) protein shares low stability and oligomerization with p53 (171). In addition, the polymerization of the splicing factor proline and glutamine (SFPQ) was shown to be essential for the cellular functions of this tumor suppressor (172) linking aggregation not only to the pathological aspects of cancer but also to functional roles of cellular nucleic acid metabolism.

**Figure 2:**
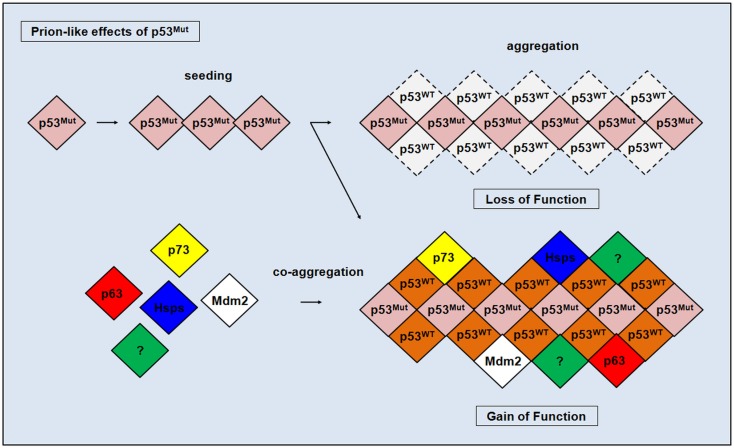
**Prion-like effects of mutated p53**. The seeding of mutated p53 (p53^Mut^) accelerates the amyloidogenic aggregation of wild-type p53 (p53^WT^) and may result in the co-aggregation of different cellular partners including p53 homologs p63 and p73, heat shock proteins (Hsps), and the p53 regulator Mdm2, and may also include additional proteins (?) that have yet to be discovered. Aggregation leads p53 to lose or gain oncogenic function.

The thermodynamics of wt and mutant p53 (R175H, C242S, R248Q, R249S, and R273H) were explored at the end of the 1990s through biophysical techniques and revealed irreversible denaturation and aggregation under certain conditions for the wt and studied mutants (173). In 2003, our group designed a study to examine the aggregation of the p53 core domain (174–176). More recently, we explored whether the wt and the p53 hot-spot mutant R248Q aggregates like an amyloid under physiological conditions and whether mutant p53 can seed the aggregation of wt p53 (22). Using a cohort of structural and cellular approaches, we established the amyloid nature of wt and mutant p53 aggregation. We showed that a seed of amyloid oligomers formed from the p53 hot-spot mutant R248Q accelerated the aggregation of wt p53 into an inactive conformation (22). We showed that a prion-like behavior of p53 would explain the dominant-negative and gain-of-function effects of mutant p53 (Figure 2). Recently, Fersht showed that different p53 mutants aggregate through a complex order mechanism and that co-aggregation can occur with wt p53 and p63 or p73 (177, 178). To address the role of aggregated p53 and the prion-like effect in triggering cancer development and progression, we observed a greater extent of mutant p53 co-aggregation with amyloid oligomers in breast cancer MDA-MB-231 cells compared with wt p53 cells (MCF7) (22). In line with our observations, similar results were observed in the biopsies of breast cancer patients carrying specific p53 mutations (R175H, H193L, I195L, Y234C, G245S, and R248Q) (29) and in the skin tissue of six patients with basal cell carcinoma (179). Furthermore, prostate cancer tissues revealed high levels of p53 immunostaining within aggregates containing mutant and wt p53 (180). Finally, cancer stem cells from a unique population of high-grade serous ovarian carcinoma (HGSOC) revealed that p53 aggregation is associated with its inactivation and platinum resistance. During differentiation to their chemosensitive progeny, these cells lost their tumor-initiating capacity and p53 aggregates (181). Moreover, using two-dimensional gel electrophoresis and mass spectrometry, these authors discovered that aggregated p53 works uniquely by interacting with proteins involved in cancer cell survival and tumor progression (181). Altogether, based on this recent *ex vivo* evidence, the involvement of p53 aggregation in cancer appears to be undisputed. Although a prion-like mechanism would explain the dominant-negative and gain-of-function effects during p53 aggregation, several questions are still awaiting exploration to define it as an etiological factor for cancer pathogenesis, invasiveness and metastasis (182).

Prions are transmissible polypeptide particles that undergo a conformational change from their cellular form (PrP^C^) to a β-sheet rich form (PrP^Sc^). This conformational modification is associated with the transmissible characteristics and pathogenesis of several diseases, including bovine spongiform encephalopathy and Creutzfeldt–Jakob disease in humans (183, 184). A genuine prion should be transmissible *in cell* and *in vivo*. Features linking p53 to prions are the conformational conversion of p53 during tumorigenesis (wt p53 to mutated p53) and the ability of mutant p53 to sequester wt p53 into amyloid species. Another interesting shared feature of p53 and PrP is their nucleic acid binding ability (23, 185–187). Although there are common characteristics between p53 and prions, no transmissible mechanisms for p53 have yet been demonstrated (182). Controversially, recent works have independently shown mechanisms of p53 secretion and uptake by cells (188–190), yielding the possibility that p53 is a transmissible particle. The Kirsten-Ras (K-Ras) oncogene protein has been shown to participate in p53 suppression by inducing Snail, and the depletion of Snail is able to induce p53 in K-Ras-mutated cancer cells but not in wt cancer cells (191, 192). Therefore, a direct correlation between K-Ras-mediated p53 suppression and tumorigenesis has been established in lung and pancreatic cancers, which have a higher frequency of K-Ras mutations (193). p53 suppression in response to the K-Ras oncogene occurs through a Snail-dependent mechanism in which p53 is secreted from cells and then taken up by K-Ras-mutated cells in caveolin-1-mediated endocytosis (189). Moreover, full-length p53 aggregates have been shown to penetrate HeLa and NIH3T3 cells via macropinocytosis and induce the aggregation of intracellular p53 (190). Although mouse models are still lacking, these surprising *in cell* transmissibility mechanisms of p53 have prompted new discussions about our proposed prion-like mechanism and have instigated ongoing experiments by our group and others to demonstrate the involvement of p53 aggregates in cancer pathogenesis and progression.

p53 aggregation and gain-of-function effects can be minimized by small molecule intervention. For instance, CDB3 is able to rescue the conformation of unstable p53 mutants (194), allowing an increase in the protein half-life to reach the nucleus and act as a tumor suppressor. In the case of PRIMA-1, its conversion to compounds that form adducts with thiols in mutant p53 is sufficient to induce apoptosis in tumor cells (195). Furthermore, resveratrol has been shown to inhibit carcinogenesis through the induction of p53-dependent cell death. Indeed, the transient transfection of wt p53 allowed H1299 cells to become more sensitive to the pro-apoptotic effects of resveratrol (196). Efforts to increase health quality are based on treatment, diagnosis and prevention. The existence of a direct correlation between the prion-like effect of aggregated p53 and tumorigenesis remains an open question, but we believe that it may provide the basis for new therapeutic interventions, early diagnosis, and cancer prevention.

## Impact of Unfolded c-Abl Core Segments on Its Functions and Activity Regulation

**Figure 3:**
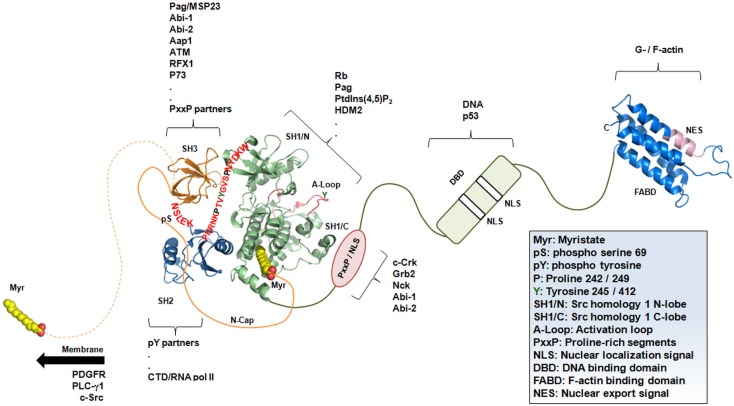
**c-Abl architecture is complex and not fully understood**. Schematic representation of full-length c-Abl showing crystal structures of the core (PDB ID: 1OPK), F-actin-binding domain – FABD (PDB ID: 1ZZP), and flexible segments at the N- (N-Cap) and C-terminal regions (proline–any residue–any residue–Proline, i.e., PxxP motifs, nuclear localization signals – NLS, and DNA-binding domain – DBD). The linker regions in the core are colored red. The cellular partners of specific domains are shown in brackets. The myristate switch (sphere representation) is presented in the open (dashed orange line) and closed (solid orange line) states.

The proper phosphorylation control of specific serine, threonine, or tyrosine residues is a fundamental step in cellular growth, survival, and death and is achieved through the tight regulation of protein kinases. As shown previously, tumor suppressors have the opposite effects. Under several types of stress, their fundamental role is to maintain genome integrity, and this is achieved by activating or repressing the transcription of specific genes to undergo biological processes, including the induction of apoptosis and growth arrest. Because kinase activity is involved in gene expression, metabolic pathways, cell growth and differentiation, membrane transport, and apoptosis, their activity must be tightly regulated. There are 518 human kinase sequences (1.7% of the entire genome), and the tyrosine kinase family is the largest with 90 members. This group of kinases is divided into 58 receptor tyrosine kinases (RTKs) and 32 non-receptor tyrosine kinases (nRTKs) (197).

c-Abl belongs to the nRTK group and has a modular and complex architecture flanked by unfolded segments (Figure 3). The proper dynamics of these segments play an important role during kinase activation/inhibition and cellular localization. The c-Abl core is composed of in-tandem Src homology (SH) 3 and SH2 domains that negatively regulate SH1 kinase activity. The SH3–SH2–SH1 domains are flanked by disordered regions at the most N-terminal region of the SH3 domain, which comprises the N-Cap region (residues 1–83), the short linker (residues 139–143: NSLEK) connecting the SH3–SH2 domains, and a longer linker between the SH2–SH1 domains (residues 237–254: PKRNKPTVYGVSPNYDKW).

Pivotal studies have clarified the participation of SH3–SH2 domains in activity regulation and oncogenic signaling of c-Abl (198–200). The presence of the SH3 domain has been shown to inhibit the transforming activity of c-Abl *in vivo* but has no effect on the *in vitro* activity, suggesting that cellular inhibitors may act in kinase inhibition (200). Not only mutations or depletions within the c-Abl SH3 domain but also the phosphorylation of a specific SH3 Tyr residue (Tyr89) are able to disrupt its negative regulatory effect (201). In the 1990s, the consensus SH3-binding site was identified as a proline-rich segment (202). In the case of c-Abl, an intramolecular SH3-domain interaction is able to regulate c-Abl activity through SH3 binding to the longer linker connecting SH2 to SH1 (203, 204). Although cellular partners have been shown to inhibit c-Abl activity (205), including retinoblastoma (Rb) protein (206), Abl-interactor proteins 1 and 2 (207, 208), ABL-associated protein-1 (209), the 23-kD macrophage stress protein MSP23 or PAG (210), F-actin (211), and BRCA1 protein (212), Superti-Furga’s group provided a basis for understanding the auto-inhibition mechanism of the c-Abl protein through the N-terminal Cap. These researchers concluded that an SH3 domain-dependent cellular inhibitor is dispensable (213). Furthermore, two consecutive crystallographic studies conducted by Superti-Furga and Kuriyan’s group extended the understanding of c-Abl auto-inhibition. These studies revealed that during inhibition, a myristoyl/phosphotyrosine switch regulates the docking and accessibility of the SH2 domain to the C-lobe of the kinase domain (KD) and that c-Abl can be activated by phosphotyrosine ligands through binding to a specific motif in the SH2 domain (FLVR motif) (214, 215).

A second crystal structure of the c-Abl core revealed more about the participation of the N-Cap segment for c-Abl inhibition. The phosphorylation of Ser69 in the N-Cap mediates interactions with the shorter linker connecting SH3 and SH2 (NSLEK region), showing that this disordered N-terminal region not only participates in presenting the myristoyl group to the kinase but also may work as a stabilizer (216). More recently, our group proposed a cascade-like mechanism for c-Abl inhibition. In this mechanism, N-Cap–SH2 interactions monitored by chemical shift perturbation analysis would provide guidance for myristate binding in the C-terminal pocket of SH1 (217). Although no unanimous data exist concerning the real c-Abl inhibitory mechanism (cellular inhibitors or auto-inhibition), functional and structural characteristics make an autoinhibitory mechanism more favored and accepted. In contrast, in a perspective paper published in Nature Cell Biology, Wang proposed a speculative model of c-Abl co-inhibition that accommodates both inhibitory mechanisms (218). It is likely that both mechanisms may influence c-Abl inhibition depending on the cellular context.

In c-Src, a prominent family of kinases composed of nine members (c-Src, Yes, Fyn, Hck, Lck, Lyn, Blk, Fgr, and Yrk), a phosphotyrosine residue in the C-terminal region of the kinase (pY527) interacts with the SH2 domain (219–221) and is considered a surrogate mechanism of the N-Cap-myristoyl group. Controversially, a c-Src crystal structure revealed a similar pocket for myristate binding (222), and more recently, membrane binding and myristoylation have been shown to regulate stability and kinase activity (223). In the case of c-Abl, transiently transfected wt and variant forms lacking myristoyl have been detected by immunoblots in crude membrane-enriched fractions (215), showing that membrane targeting may be a multifactorial event. One possible surrogate for myristate, as an inhibitor molecule, is phosphatidylinositol-4,5-biphosphate [PtdIns (4,5)P_2_], the product of PLC-γ1 (224). In the case of c-Src, myristoylation is assisted by a polybasic cluster of amino acids that tightly bind the kinase to membrane regions (225, 226). These regulatory differences between c-Src and c-Abl kinase members reveal the complexity and plasticity of tyrosine-related kinases (227).

Small-angle X-ray scattering studies of an activated c-Abl form, in which the N-Cap region is depleted and two additional mutations are introduced (P242E/P249E), revealed an elongated shape consistent with the SH2 domain sitting at the top of the SH1 N-lobe (216). This SH2-kinase intramolecular interaction has been shown to be necessary for Bcr–Abl catalytic activity and was validated as an allosteric target for therapeutic intervention because its disruption completely abolished leukemia formation in mice (228). Altogether, these data suggest that, upon complete activation, the N-Cap-myristoyl tether would detach from the SH1 C-terminal pocket. However, a theoretical model proposed that the equilibrium fraction of c-Abl in which myristoyl is unlatched is only ~0.5% (229). An intermolecular autophosphorylation of Tyr412, located at the activation loop, followed by the phosphorylation of Tyr245, which is located in the longer linker between the SH2–SH1 domains, also participates in enzyme activation (230). To understand the possible role of a detached N-Cap-myristoyl tether inside the cell, our group asked whether myristoylated c-Abl would bind to the cellular membrane. Although c-Abl was initially shown to be present in pseudopodia protrusions, after stimulation with hepatocyte growth factor (HGF) in thyroid cancer cells (231), we demonstrated that the N-Cap-myristoyl tether may play a role in protein inhibition and also may direct the c-Abl protein to anchor in the membrane as an additional mechanism to stabilize this disordered segment, which may also be linked to early apoptotic signaling (217). Although we proposed a link to apoptosis during transport to the membrane, the membrane pool of c-Abl is also linked to cytoskeletal reorganization (232, 233), cell migration, and neurite outgrowth. For example, c-Abl activation downstream to the platelet-derived growth factor receptor (PDGFR) has been shown to require functional phospholipase C-γ1 (PLC-γ1), creating a bidirectional link between PLC-γ1 and c-Abl in the membrane ruffling signaling pathway (224).

## The Impact of Unfolded C-Terminal Segments of c-Abl on Its Nuclear Functions

c-Abl kinase is not exclusive in the broad spectrum of biological functions triggered by its activity because neonatal lethality was observed in c-Abl^−/−^ mice and in mice carrying a truncated form of the c-Abl in which the C-terminal region was depleted (234, 235). The C-terminal region of c-Abl is composed of more than 600 residues with several flexible and disordered segments containing three nuclear localization signals (NLSs) (236, 237), an NES (238) with multiple proline-rich motifs and multiple protein-binding sites; a DBD (239) and a structured F-actin-binding domain (FABD) (Figure 3). In addition to the FABD (240), structural information from this C-terminal region is still lacking probably because of its increased flexibility and the presence of non-crystallizable segments. Indeed, the DBD of c-Abl does not bind DNA with a high degree of sequence specificity (241), and a computational prediction of its potential secondary structure revealed that it is mainly formed of coil conformations (242).

**Figure 4:**
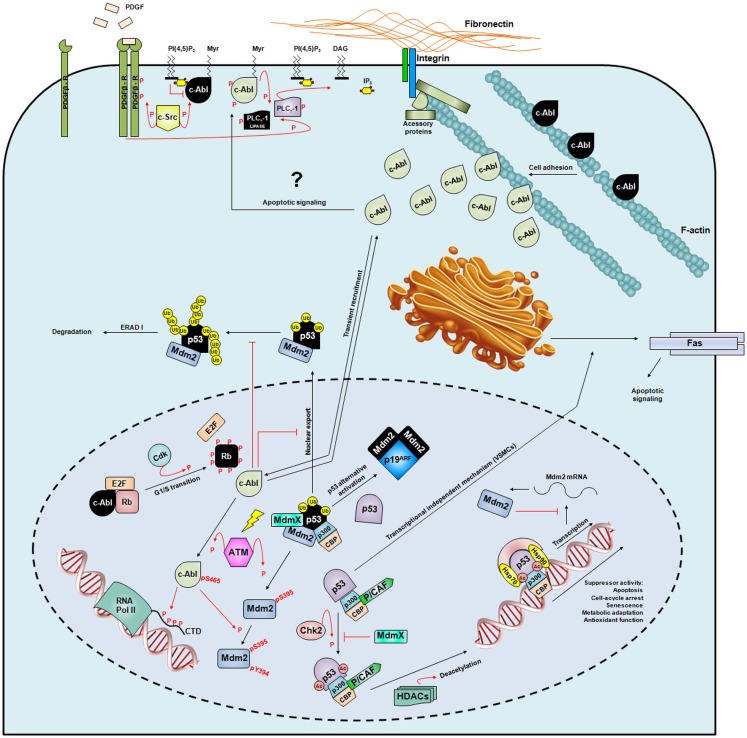
**Intracellular signaling of c-Abl**. A schematic representation of the broad spectrum of c-Abl signaling pathways in different sub-cellular compartments. The nuclei are shown by dashed lines. Inactivated c-Abl, Rb, p53, Mdm2, and PLC_γ_-1 proteins are colored black to distinguish them from activated forms (represented in different colors). “P” in red means phosphorylation, “Ub” in yellow ubiquitination, “Ac” in light red acetylation, and the yellow ray is genotoxic stress. Red and black arrows represent signaling through phosphorylation and promoting activity, respectively, and red lines with a crossbar indicate signaling inhibition. Molecules anchored to the membrane are: PI(4,5)P_2_ – phosphatidylinositol-4,5-biphosphate, Myr – myristate, and DAG – diacylglycerol.

The presence of NLS and NES confers to the protein the ability to shuttle between these two cellular compartments depending on the cellular requirements (Figure 4). The nuclear c-Abl pool is involved in the control of cell-cycle-dependent and DNA damage-induced gene expression. The main regulator of nuclear c-Abl is the retinoblastoma (Rb) tumor suppressor, which binds through its C-terminal tail to the c-Abl ATP-binding lobe (206). In resting G1 cells, Rb is found unphosphorylated, and this is the active state for its growth-inhibitory activity. Upon G1/S transition, Rb becomes hyperphosphorylated by cdk/cyclin kinases (243) and releases the Rb/c-Abl complex, allowing c-Abl to become activated. The assembly of Rb-mediated complexes, such as the ternary complex of E2F/Rb/c-Abl, has been shown to be important for cell-cycle arrest (244). Moreover, the cytostatic effect of c-Abl (245) has been shown to be dependent not only on Rb but also on p53 (237) (Figure 4). However, Sawyer’s group observed that growth suppression triggered by c-Abl requires p53 but not Rb (246).

The 52 tandem heptapeptide repeats (YSPTSPS) in the carboxyl-terminal domain (CTD) of RNA polymerase II are extensively phosphorylated on serine, threonine, and tyrosine residues as well as by O-linked glycosylation (247). The RNA pol II CTD tail has been shown to be a specific phosphorylation target for nuclear activated c-Abl but not c-Src (248). CTD phosphorylation by c-Abl requires an active c-Abl SH2 domain that binds to partially phosphorylated CTD and increases processivity (249), but a second c-Abl CTD-interacting domain at the c-Abl C-terminal region is also required (250).

Nuclear c-Abl is activated through phosphorylation on Ser465 by ataxia telangiectasia-mutated (ATM) kinase after ionizing radiation (251, 252), which also phosphorylates the major p53-negative regulator Hdm2 on Ser395 (253, 254) (Figure 4). Activated c-Abl phosphorylates Hdm2 on Tyr394 (255), and through this phosphate-exchanging mechanism, Hdm2 impairs its inhibitory activity toward p53, which becomes active in the nucleus (256, 257). Although c-Abl and p53 are both activated upon genotoxin exposure (258), it appears that only p53 is considered a universal sensor of genotoxic stress (259). Depending on the extent of DNA damage, p53 signaling can induce the transcription of genes involved in at least three processes that govern cell fate: cell-cycle arrest, cellular senescence, and apoptosis (260).

## The Impact of Unfolded c-Abl Segments on Its Cytoplasmic Functions and Cellular Localization

We have recently shown that the N-Cap and SH3 domains of c-Abl acquire microsecond–millisecond motions upon N-Cap association with the SH2-L and that the N-Cap-myristoyl tether likely triggers the protein to anchor to the membrane in a function- and stability-dependent mechanism (217). Although membrane anchoring is linked to early apoptotic signaling, the membrane pool of c-Abl is also involved in biological processes, such as membrane ruffling, mitogenesis, and chemotaxis. Upon PDGF stimulation, PDGFRs dimerize and recruit c-Src and PLC-γ1 to activate membrane-bound c-Abl (261). Moreover, c-Abl and the Abl-related gene (Arg) have been shown to form an inducible complex with PDGFR and change phosphoryl groups (262). After activation, c-Abl binds to PLC-γ1 and inhibits its lipase activity through phosphorylation (224, 261).

The cytosolic pool of c-Abl is able to bind the F-actin network (263), and upon binding, its kinase activity is abolished (211) (Figure 4). A proline-rich region in the C-terminus of c-Abl has also been shown to sequester G-actin and bundle F-actin filaments *in vitro* (263), a mechanism that has also been observed for Arg (264). The detachment of cells from the extracellular matrix (ECM) does not influence the kinase activity of the nuclear and cytoplasmic c-Abl pools, but during cell adhesion to fibronectin, c-Abl becomes activated, and the nuclear pool is transiently recruited to the cytosol. These results suggest that c-Abl can induce integrin signaling to the nucleus to coordinate adhesion and cell-cycle signals (265). During the cell cycle, the cdc2 Ser/Thr kinase is required for G1/S and G2/M transitions and is responsible for the hyperphosphorylation of the c-Abl C-terminal region, which may participate in cell-cycle regulation (239). Altogether, the one-gene-one-function paradigm is beyond the c-Abl biological roles. Because of its intrinsic complexity and flexibility, more than 20 years of research have been required to begin deciphering its physiological functions, and it is unclear how many more years will be needed to completely understand this enigmatic and hard-working kinase.

## The Involvement of c-Abl in Leukemia and Solid Tumors

Chronic myeloid leukemia is a biphasic disease with a chronic phase ranging from 3 to 4 years that is characterized by a massive expansion of granulocytic cells. This step is followed by a blast phase in which cell differentiation is blocked, leading to extramedullary infiltrates of immature myeloid or lymphoid cells (blasts) in the peripheral blood, liver, spleen, or lymph nodes. Beyond the clinical features of hematopoietic malignances, such as CML, the c-Abl oncoprotein is also involved in malignant solid tumors of the breast, lung, colon, and kidney (266–269). In these tumors, the role of c-Abl activation is not linked to Bcr–Abl translocation but is associated with gene amplification, protein overexpression, oncogenic tyrosine kinases, chemokine receptors, oxidative stress, and negative regulatory protein inhibition (270, 271).

The discovery of the oncogenic effect of the c-Abl protein originates from early studies of the Moloney leukemia virus (MLV), which triggers a thymic-independent lymphatic neoplasm upon inoculation in mice (272, 273). The discovery of a small, abnormal chromosome named the Philadelphia chromosome (Ph) was the first consistent chromosomal abnormality associated with a specific type of leukemia (274). In 1973, this shortened chromosome was characterized as the reciprocal translocation t(9;22) through quinacrine fluorescence and Giemsa staining (275). The Ph chromosome is found in the myeloid, erythroid, megakaryocytic, and B lymphoid lineages, indicating that it represents a stem cell-proliferative disorder.

At the molecular level, Ph translocation creates a hybrid gene known as *bcr–abl* that encodes three main protein isoforms (p190^Bcr–Abl^, p210^Bcr–Abl^, or p230^Bcr–Abl^) with different leukemic phenotypes (69). Genomic breaks occur within the break cluster region (*bcr*) gene at three different regions classified as minor-bcr, major-bcr, and micro-bcr (276). In the *c-abl* gene, a non-random break also occurs within the first intron (277), and this break removes the residues corresponding to the N-Cap-myristoyl tether. As a result, the chimeric Bcr–Abl loses the pivotal N-Cap-myristoyl inhibitory mechanism and becomes highly active. The *bcr–abl* fusion is considered a hallmark of CML pathogenesis. Because the anchoring of c-Abl to the membrane through the N-Cap-myristate tether is linked to pro-apoptotic signaling (217) and Bcr–Abl is exclusively cytosolic (278), we believe that this “missing link” in the Bcr–Abl chimera may help explain the apoptotic resistance phenotype of Bcr–Abl-positive cells. Through the activation of the transcription factor STAT5, Bcr–Abl specifically increases the expression of the antiapoptotic proteins Bcl-2 and Bcl-X (279–281). Additionally, Bcr–Abl has been shown to prevent mitochondrial cytochrome *c* release through the inhibition of Bad by phosphatidylinositol 3-kinase/Akt-dependent signaling (282). Moreover, the mechanism explaining the antiapoptotic mechanism of Bcr–Abl has been shown to occur downstream of mitochondrial cytochrome *c* release, preventing the binding of Apaf-1 to caspase 9 (283). The involvement of a membrane-bound c-Abl in apoptotic signaling requires further exploration.

## c-Abl Mutations are a Consequence of a Drug-Resistance Phenotype

Different from p53, which contains mutations that are associated with dominant-negative, loss-of-function and gain-of-function mechanisms in cancer, c-Abl mutations are a consequence of a drug-resistance phenotype. The front-line and well-accepted therapy for early diagnosed chronic CML patients is 2-phenylaminopyrimidine or imatinib mesylate (284, 285), which has also been shown to be effective for blast crisis (286). Although effective, continuous treatment with this ATP-competitive Bcr–Abl inhibitor leads to patient relapse in the majority of cases due to KD-acquired mutations. However, this resistance mechanism is not exclusive (287–289). Indeed, imatinib resistance has been shown to occur without amplification or mutations in Bcr–Abl (290). Moreover, the overexpression of multidrug-resistance genes may also participate in imatinib resistance (291, 292). The exploration of a drug-resistance signature using microarrays has revealed the upregulation of apoptosis-related genes and the downregulation of genes involved in energy metabolism in K562 sublines established with different dosages of imatinib (293). Imatinib insensitivity is not only related to a drug-resistance phenotype. Ph-positive stem cells (CD34+) from patients with chronic phase CML are equally resistant to the drug (294) and exhibit enhanced MAP kinase activity after treatment (295). Moreover, microarrays comparing CML CD34+ cells with normal CD34+ cells have revealed Bcr–Abl-induced functional alterations, such as increased cell-cycle and proteasome activity (296). Altogether, these imatinib-insensitive stem cells likely explain the maintenance of a minimal residual disease phenotype and relapse.

Almost a year after Druker’s work, which showed drug efficacy, a series of patient follow-up studies evidenced a high frequency of point mutations within the KD ATP-binding region or within the P-loop associated with poor prognosis (297–299). Controversially, several imatinib-resistant KD mutants have been shown to remain sensitive to this drug (300). Crystallographic studies of the wt and the T315I hot-spot mutant have aided the understanding of the contacts of imatinib within the c-Abl KD (301, 302) and the mechanisms underlying resistance (303) but even the second-generation (nilotinib and dasatinib) and several third-generation ATP-competitive kinase inhibitors succumb to overcome T315I gain-of-function effects (304–306). The involvement of the myristate pocket in kinase inhibition gives rise to the targeting of Bcr–Abl through allosteric mechanisms. Although some imatinib-resistant Bcr–Abl mutants are sensitive to the myristate-like inhibitor GNF-2, T315I is not (307). However, the association of nilotinib and GNF-5, a close derivative of GNF-2, was able to prolong the survival of mice in a Bcr–Abl T315I xenograft model (308). The recent idea of targeting Bcr–Abl is based on allosteric inhibitors targeting the myristate pocket, the Bcr N-terminal coiled-coil oligomerization domain (309) or the SH2–KD interaction (228). Understanding how Bcr–Abl works in a dynamic and synergistic manner will provide the missing clues to increase the repertoire of targetable segments for drug design.

## Future Directions

The growing number of proteins with in-tandem structured and unstructured regions will challenge the next generation of researchers to uncover the mechanisms underlying their intramolecular dynamic synergism. Several of these unstructured segments located at the N- or C-termini or between structured regions were previously not thought to participate in protein activity, regulation, cancer initiation, and abrogation. This scenario is currently changing due to a combination of 10 years of efforts in cellular and molecular signaling and the use of atomic and sub-atomic methods, such as small-angle X-ray scattering (310) and NMR. It is now possible to reconstruct models of partially structured domains and flexible linkers at sub-atomic resolution, as shown for the quaternary structures of full-length p53 in a DNA-bound complex (311, 312), and also to estimate the frequency of the conformational distribution for an ensemble of totally unstructured segments. These methods are complementary to atomic resolution methods that are at the limit of providing information from disordered regions. More than 30% of eukaryotic genomes encode unfolded regions with more than 30 residues, and this number increases to 80% in cancer-associated proteins. The challenge for the next era will be to reveal the hierarchical interactome of these very flexible proteins and to understand how they act synergistically to promote homeostasis and tumorigenesis.

## Conflict of Interest Statement

The authors declare that the research was conducted in the absence of any commercial or financial relationships that could be construed as a potential conflict of interest.

## References

[1] Akasaka K Probing conformational fluctuation of proteins by pressure perturbation. Chem Rev (2006) 106(5):1814–35 10.1021/cr040440z 16683756

[2] Akasaka K Kitahara R Kamatari YO. Exploring the folding energy landscape with pressure. Arch Biochem Biophys (2013) 531(1–2):110–5. 10.1016/j.abb.2012.11.016 23246376

[3] Silva JL Oliveira AC Vieira TC de Oliveira GA Suarez MC Foguel D High-pressure chemical biology and biotechnology. Chem Rev (2014) 114(14):7239–67 10.1021/cr400204z 24884274

[4] Dzwolak W Kato M Taniguchi Y. Fourier transform infrared spectroscopy in high-pressure studies on proteins. Biochim Biophys Acta (2002) 1595(1–2):131–44. 10.1016/S0167-4838(01)00340-5 11983392

[5] Winter R. Synchrotron X-ray and neutron small-angle scattering of lyotropic lipid mesophases, model biomembranes and proteins in solution at high pressure. Biochim Biophys Acta (2002) 1595(1–2):160–84. 10.1016/S0167-4838(01)00342-9 11983394

[6] Lerch MT Horwitz J McCoy J Hubbell WL. Circular dichroism and site-directed spin labeling reveal structural and dynamical features of high-pressure states of myoglobin. Proc Natl Acad Sci U S A (2013) 110(49):E4714–22. 10.1073/pnas.1320124110 24248390 PMC3856799

[7] Onuchic JN Luthey-Schulten Z Wolynes PG. Theory of protein folding: the energy landscape perspective. Annu Rev Phys Chem (1997) 48:545–600. 10.1146/annurev.physchem.48.1.545 9348663

[8] Ellgaard L Helenius A Quality control in the endoplasmic reticulum. Nat Rev Mol Cell Biol (2003) 4(3):181–91 10.1038/nrm1052 12612637

[9] Fewell SW Travers KJ Weissman JS Brodsky JL. The action of molecular chaperones in the early secretory pathway. Annu Rev Genet (2001) 35:149–91. 10.1146/annurev.genet.35.102401.090313 11700281

[10] Ghaemmaghami S Huh WK Bower K Howson RW Belle A Dephoure N Global analysis of protein expression in yeast. Nature (2003) 425(6959):737–41 10.1038/nature02046 14562106

[11] Hendershot LM. Giving protein traffic the green light. Nat Cell Biol (2000) 2(6):E105–6. 10.1038/35014100 10854339

[12] van Galen P Kreso A Mbong N Kent DG Fitzmaurice T Chambers JE The unfolded protein response governs integrity of the haematopoietic stem-cell pool during stress. Nature (2014) 510(7504):268–72. 10.1038/nature13228 24776803

[13] Walter P Ron D. The unfolded protein response: from stress pathway to homeostatic regulation. Science (2011) 334(6059):1081–6. 10.1126/science.1209038 22116877

[14] Hetz C Martinon F Rodriguez D Glimcher LH. The unfolded protein response: integrating stress signals through the stress sensor IRE1α. Physiol Rev (2011) 91(4):1219–43. 10.1152/physrev.00001.2011 22013210

[15] Hetz C. The unfolded protein response: controlling cell fate decisions under ER stress and beyond. Nat Rev Mol Cell Biol (2012) 13(2):89–102. 10.1038/nrm3270 22251901

[16] Sitia R Braakman I. Quality control in the endoplasmic reticulum protein factory. Nature (2003) 426(6968):891–4. 10.1038/nature02262 14685249

[17] Ismail N Ng DT. Have you HRD? Understanding ERAD is DOAble! Cell (2006) 126(2):237–9. 10.1016/j.cell.2006.07.001 16873052

[18] Meusser B Hirsch C Jarosch E Sommer T. ERAD: the long road to destruction. Nat Cell Biol (2005) 7(8):766–72. 10.1038/ncb0805-766 16056268

[19] Römisch K. Endoplasmic reticulum-associated degradation. Annu Rev Cell Dev Biol (2005) 21:435–56. 10.1146/annurev.cellbio.21.012704.133250 16212502

[20] Kawaguchi K Ng DTW SnapShot: ER-associated protein degradation pathways. Cell (2007) 129(6):1230.e1–e2 10.1016/j.cell.2007.06.005 17574032

[21] Chiti F Dobson CM. Protein misfolding, functional amyloid, and human disease. Annu Rev Biochem (2006) 75:333–66. 10.1146/annurev.biochem.75.101304.123901 16756495

[22] Ano Bom AP Rangel LP Costa DC de Oliveira GA Sanches D Braga CA Mutant p53 aggregates into prion-like amyloid oligomers and fibrils: implications for cancer. J Biol Chem (2012) 287(33):28152–62. 10.1074/jbc.M112.340638 22715097 PMC3431633

[23] Silva JL Vieira TC Gomes MP Bom AP Lima LM Freitas MS Ligand binding and hydration in protein misfolding: insights from studies of prion and p53 tumor suppressor proteins. Acc Chem Res (2010) 43(2):271–9. 10.1021/ar900179t 19817406 PMC2825094

[24] Silva JL Rangel LP Costa DC Cordeiro Y De Moura Gallo CV. Expanding the prion concept to cancer biology: dominant-negative effect of aggregates of mutant p53 tumor suppressor. Biosci Rep (2013) 33(4):e00054. 10.1042/BSR20130065 24003888 PMC3728989

[25] Kandoth C McLellan MD Vandin F Ye K Niu B Lu C Mutational landscape and significance across 12 major cancer types. Nature (2013) 502(7471):333–9. 10.1038/nature12634 24132290 PMC3927368

[26] Coffill CR Muller PA Oh HK Neo SP Hogue KA Cheok CF Mutant p53 interactome identifies nardilysin as a p53R273H-specific binding partner that promotes invasion. EMBO Rep (2012) 13(7):638–44. 10.1038/embor.2012.74 22653443 PMC3388785

[27] Trinidad AG Muller PA Cuellar J Klejnot M Nobis M Valpuesta JM Interaction of p53 with the CCT complex promotes protein folding and wild-type p53 activity. Mol Cell (2013) 50(6):805–17. 10.1016/j.molcel.2013.05.002 23747015 PMC3699784

[28] Rangel LP Costa DC Vieira TC Silva JL. The aggregation of mutant p53 produces prion-like properties in cancer. Prion (2014) 8(1):75–84. 10.4161/pri.27776 24509441 PMC7030899

[29] Levy CB Stumbo AC Ano Bom AP Portari EA Cordeiro Y Silva JL Co-localization of mutant p53 and amyloid-like protein aggregates in breast tumors. Int J Biochem Cell Biol (2011) 43(11):60–4. 10.1016/j.biocel.2010.10.017 21056685

[30] Bom AP Freitas MS Moreira FS Ferraz D Sanches D Gomes AM The p53 core domain is a molten globule at low pH: functional implications of a partially unfolded structure. J Biol Chem (2010) 285(4):2857–66. 10.1074/jbc.M109.075861 19933157 PMC2807339

[31] Park SJ Borin BN Martinez-Yamout MA Dyson HJ. The client protein p53 adopts a molten globule-like state in the presence of Hsp90. Nat Struct Mol Biol (2011) 18(5):537–41. 10.1038/nsmb.2045 21460846 PMC3087862

[32] Sevier CS Kaiser CA. Formation and transfer of disulphide bonds in living cells. Nat Rev Mol Cell Biol (2002) 3(11):836–47. 10.1038/nrm954 12415301

[33] Bertolotti A Zhang Y Hendershot LM Harding HP Ron D. Dynamic interaction of BiP and ER stress transducers in the unfolded-protein response. Nat Cell Biol (2000) 2(6):326–32. 10.1038/35014014 10854322

[34] Okamura K Kimata Y Higashio H Tsuru A Kohno K. Dissociation of Kar2p/BiP from na ER sensory molecule, Ire1p, triggers the unfolded protein response in yeast. Biochem Biophys Res Commun (2000) 279(2):445–50. 10.1006/bbrc.2000.3987 11118306

[35] Haze K Yoshida H Yanagi H Yura T Mori K. Mammalian transcription factor ATF6 is synthesized as a transmembrane protein and activated by proteolysis in response to endoplasmic reticulum stress. Mol Biol Cell (1999) 10(11):3787–99. 10.1091/mbc.10.11.3787 10564271 PMC25679

[36] Shen J Chen X Hendershot L Prywes R. ER stress regulation of ATF6 localization by dissociation of BiP/GRP78 binding and unmasking of Golgi localization signals. Dev Cell (2002) 3(1):99–111. 10.1016/S1534-5807(02)00203-4 12110171

[37] Shiu RP Pouyssegur J Pastan I. Glucose depletion accounts for the induction of two transformation-sensitive membrane proteinsin Rous sarcoma virus-transformed chick embryo fibroblasts. Proc Natl Acad Sci U S A (1977) 74(9):3840–4. 10.1073/pnas.74.9.3840 198809 PMC431753

[38] Warburg O On respiratory impairment in cancer cells. Science (1956) 124(3215):269–70. 13351639

[39] Warburg O On the origin of cancer cells. Science (1956) 123(3191):309–14 10.1126/science.123.3191.309 13298683

[40] Wang W Groenendyk J Michalak M. Endoplasmic reticulum stress associated responses in cancer. Biochim Biophys Acta (2014) 1843(10):2143–9. 10.1016/j.bbamcr.2014.01.012 24440276

[41] Li J Lee AS. Stress induction of GRP78/BiP and its role in cancer. Curr Mol Med (2006) 6(1):45–54. 10.2174/156652406775574523 16472112

[42] Fernandez PM Tabbara SO Jacobs LK Manning FC Tsangaris TN Schwartz AM Overexpression of the glucose-regulated stress gene GRP78 in malignant but not benign human breast lesions. Breast Cancer Res Treat (2000) 59(1):15–26. 10.1023/A:1006332011207 10752676

[43] Xing X Lai M Wang Y Xu E Huang Q. Overexpression of glucose-regulated protein 78 in colon cancer. Clin Chim Acta (2006) 364(1–2):308–15. 10.1016/j.cca.2005.07.016 16182273

[44] Dong D Stapleton C Luo B Xiong S Ye W Zhang Y A critical role for GRP78 in the tumor microenvironment for neovascularization during tumor growth and metastasis. Cancer Res (2011) 71(8):2848057. 10.1158/0008-5472.CAN-10-3151 21467168 PMC3078191

[45] Dong D Ni M Li J Xiong S Ye W Virrey JJ Critical role of the stress chaperone GRP78/BiP in tumor proliferation, survival, and tumor angiogenesis in transgene-induced mammary tumor development. Cancer Res (2008) 68(2):498–505. 10.1158/0008-5472.CAN-07-2950 18199545

[46] Li Z Li Z. Glucose regulated protein 78: a critical link between tumor microenvironment and cancer hallmarks. Biochim Biophys Acta (2012) 1826(1):13–22. 10.1016/j.bbcan.2012.02.001 22426159

[47] Misra UK Payne S Pizzo SV. Ligation of prostate cancer cell surface GRP78 activates a proproliferative and antiapoptotic feedback loop: a role for secreted prostate-specific antigen. J Biol Chem (2011) 286(2):1248–59. 10.1074/jbc.M110.129767 21056970 PMC3020732

[48] Nagaoka T Karasawa H Castro NP Rangel MC Salomon DS Bianco C Na evolving web of signaling networks regulated by Cripto-1. Growth Factors (2012) 30(1):13–21 10.3109/08977194.2011.641962 22149969

[49] Nakamura S Takizawa H Shimazawa M Hashimoto Y Sugitani S Tsuruma K. Mild endoplasmic reticulum stress promotes retinal neovascularization via induction of BiP/GRP78. PLoS One (2013) 8(3):e60517. 10.1371/journal.pone.0060517 23544152 PMC3609792

[50] Davidson DJ Haskell C Majest S Kherzai A Egan DA Walter KA Kringle 5 of human plasminogen induces apoptosis of endothelial and tumor cells through surface-expressed glucose-regulated protein 78. Cancer Res (2005) 65(11):4663–72. 10.1158/0008-5472.CAN-04-3426 15930284

[51] Fu Y Li J Lee AS. GRP78/BiP inhibits endoplasmic reticulum BIK and protects human breast cancer cells against estrogen starvation-induced apoptosis. Cancer Res (2007) 67(8):3734–40. 10.1158/0008-5472.CAN-06-4594 17440086

[52] Reddy RK Mao C Baumeister P Austin RC Kaufman RJ Lee AS. Endoplasmic reticulum chaperone protein GRP78 protects cells from apoptosis induced by topoisomerase inhibitors: role of ATP binding site in suppresion of caspase-7 activation. J Biol Chem (2003) 278(23):20915–24. 10.1074/jbc.M212328200 12665508

[53] Zhao R Davey M Hsu YC Kaplanek P Tong A Parsons AB Navigating the chaperone network: an integrative map of physical and genetic interactions mediated by the hsp90 chaperone. Cell (2005) 120(5):715–27. 10.1016/j.cell.2004.12.024 15766533

[54] Trepel J Mollapour M Giaccone G Neckers L. Targeting the dynamic HSP90 complex in cancer. Nat Rev Cancer (2010) 10(8):537–49. 10.1038/nrc2887 20651736 PMC6778733

[55] Ci W Polo JM Cerchietti L Shaknovich R Wang L Yang SN The BCL6 transcriptional program features repression of multiple oncogenes in primary B cells and is deregulated in DLBCL. Blood (2009) 113(22):5536–48. 10.1182/blood-2008-12-193037 19307668 PMC2689052

[56] Cerchietti LC Lopes EC Yang SN Hatzi K Bunting KL Tsikitas LA A purine scaffold Hsp90 inhibitor destabilizes BCL-6 and has specific antitumor activity in BCL-6-dependent B cell lymphomas. Nat Med (2009) 15(12):1369–76. 10.1038/nm.2059 19966776 PMC2805915

[57] Maki CG Huibregtse JM Howley PM. In vivo ubiquitination and proteasome-mediated degradation of p53(1). Cancer Res (1996) 56(11):2649–54. 8653711

[58] King FW Wawrzynow A Höhfeld J Zylicz M. Co-chaperones Bag-1, Hop and Hsp40 regulate Hsc70 and Hsp90 interactions with wild-type or mutant p53. EMBO J (2001) 20(22):6297–305. 10.1093/emboj/20.22.6297 11707401 PMC125724

[59] Walerych D Kudla G Gutkowska M Wawrzynow B Muller L King FW Hsp90 chaperones wild-type p53 tumor suppressor protein. J Biol Chem (2004) 279(47):48836–45. 10.1074/jbc.M407601200 15358769

[60] Müller L Schaupp A Walerych D Wegele H Buchner J. Hsp90 regulates the activity of wild type p53 under physiological and elevated temperatures. J Biol Chem (2004) 279(47):48846–54. 10.1074/jbc.M407687200 15358771

[61] Walerych D Olszewski MB Gutkowska M Helwak A Zylicz M Zylicz A. Hsp70 molecular chaperones are required to support p53 tumor suppressor activity under stress conditions. Oncongene (2009) 28(48):4284–94. 10.1038/onc.2009.281 19749793

[62] Walerych D Zylicz A Wallace M Hupp T Zylicz M. MDM2 chaperones the p53 tumor suppressor. J Biol Chem (2007) 282(45):32603–12. 10.1074/jbc.M702767200 17848574

[63] Whitesell L Sutphin P An WG Schulte T Blagosklonny MV Neckers L. Geldanamycin-stimulated destabilization of mutated p53 is mediated by proteasome in vivo. Oncogene (1997) 14(23):2809–16. 10.1038/sj.onc.1201120 9190897

[64] Finlay CA Hinds PW Tan TH Eliyahu D Oren M Levine AJ. Activating mutations for transformation by p53 produce a gene product that forms an hsc70-p53 complex with an altered half-life. Mol Cell Biol (1988) 8(2):531–9. 2832726 10.1128/mcb.8.2.531-539.1988 PMC363177

[65] Whitesell L Sutphin PD Pulcini EJ Martinez JD Cook PH. The physical association of multiple molecular chaperone proteins with mutant p53 is altered by geldanamycin, an hsp90-binding agent. Mol Cell Biol (1998) 18(3):1517–24. 9488468 10.1128/mcb.18.3.1517 PMC108866

[66] Miller P Schnur RC Barbacci E Moyer MP Moyer JD. Binding of benzoquinoid ansamycins to p100 correlates with their ability to deplete the erbB2 gene product p185. Biochem Biophys Res Commun (1994) 201(3):1313–9. 10.1006/bbrc.1994.1847 7912926

[67] Whitesell L Mimnaugh EG De Costa B Myers CE Neckers LM. Inhibition of heat shock protein HSP90-pp60v-src heteroprotein complex formation by benzoquinone ansamycins: essential role for stress proteins in oncogenic transformation. Proc Natl Acad Sci U S A (1994) 91(18):8324–8. 10.1073/pnas.91.18.8324 8078881 PMC44598

[68] Schulte TW Blagosklonny MV Romanova L Mushinski JF Monia BP Johnston JF Destabilization of Raf-1 by geldanamycin leads to disruption of the Raf-1-MEK-mitogen-activated protein kinase signalling pathway. Mol Cell Biol (1996) 16(10):5839–45. 8816498 10.1128/mcb.16.10.5839 PMC231585

[69] Clark SS McLaughlin J Crist WM Champlin R Witte ON. Unique forms og the abl tyrosine kinase distinguish Ph1-positive CML from Ph1-positive ALL. Science (1987) 235(4784):85–8. 10.1126/science.3541203 3541203

[70] Blagosklonny MV Fojo T Bhalla KN Kim JS Trepel JB Figg WD The Hsp90 inhibitor geldanamycin selectively sensitizes Bcr-Abl-expressing leukemia cells to cytotoxic chemotherapy. Leukemia (2001) 15(10):1537–43. 10.1038/sj.leu.2402257 11587211

[71] Shiotsu Y Neckers LM Wortman I An WG Schulte TW Soga S Novel oxime derivatives of radicicol induce erythroid differentiation associated with preferential G(1) phase accumulation against chronic myelogenous leukemia cells through destabilization of Bcr-Abl with Hsp90 complex. Blood (2000) 96(6):2284–91. 10979978

[72] Wu LX Xu JH Zhang KZ Lin Q Huang XW Wen CX Disruption of the Bcr-Abl/Hsp90 protein complex: a possible mechanism to inhibit Bcr-Abl-positive human leukemic blasts by novobiocin. Leukemia (2008) 22(7):1402–9. 10.1038/leu.2008.89 18418407

[73] Gorre ME Ellwood-Yen K Chiosis G Rosen N Sawyers CL. BCR-ABL point mutants isolated from patients with imatinib mesylate-resistant chronic myeloid leukemia remain sensitive to inhibitors of the BCR-ABL chaperone heat shock protein 90. Blood (2002) 100(8):3041–4. 10.1182/blood-2002-05-1361 12351420

[74] Peng C Brain J Hu Y Goodrich A Kong L Grayzel D Inhibition of heat shock protein 90 prolongs survival of mice with BCR-ABL-T315I-induced leukemia and suppresses leukemic stem cells. Blood (2007) 110(2):678–85. 10.1182/blood-2006-10-054098 17395781 PMC1924476

[75] Kancha RK Bartosch N Duyster J. Analysis of conformational determinants underlying HSP90-kinase interaction. PLoS One (2013) 8(7):e68394. 10.1371/journal.pone.0068394 23844194 PMC3699556

[76] Hanahan D Weinberg RA Hallmarks of cancer: the next generation. Cell (2011) 144(5):646–74 10.1016/j.cell.2011.02.013 21376230

[77] Botuyan MV Momand J Chen Y. Solution conformation of an essential region of the p53 transactivation domain. Fold Des (1997) 2(6):331–42. 10.1016/S1359-0278(97)00047-3 9427007

[78] Dawson R Müller L Dehner A Klein C Kessler H Buchner J. The N-terminal domain of p53 is natively unfolded. J Mol Biol (2003) 332(5):1131–41. 10.1016/j.jmb.2003.08.008 14499615

[79] Lee H Mok KH Muhandiram R Park KH Suk JE Kim DH Local structural elements in the mostly unstructured transcriptional activation domain of human p53. J Biol Chem (2000) 275(38):29426–32. 10.1074/jbc.M003107200 10884388

[80] Lee CW Martinez-Yamout MA Dyson HJ Wright PE. Structure of the p53 transactivation domain in complex with the nuclear receptor coactivator binding domain of CREB binding domain. Biochemistry (2010) 49(46):9964–71. 10.1021/bi1012996 20961098 PMC2982890

[81] Kussie PH Gorina S Marechal V Elenbaas B Moreau L Levine AJ Structure of the MDM2 oncoprotein bound to the p53 tumor suppressor transactivation domain. Science (1996) 274(5289):948–53 10.1126/science.274.5289.948 8875929

[82] Ferreon JC Lee CW Arai M Martinez-Yamout MA Dyson HJ Wright PE. Cooperative regulation of p53 by modulation of ternary complex formation with CBP/p300 and HDM2. Proc Natl Acad Sci U S A (2009) 106(16):6591–6. 10.1073/pnas.0811023106 19357310 PMC2672497

[83] Shieh SY Ikeda M Taya Y Prives C. DNA damage-induced phosphorylation of p53 alleviates inhibition by MDM2. Cell (1997) 91(3):325–34. 10.1016/S0092-8674(00)80416-X 9363941

[84] Dumaz N Meek DW. Serine15 phosphorylation stimulates p53 transactivation but does not directly influence interaction with HDM2. EMBO J (1999) 18(24):7002–10. 10.1093/emboj/18.24.7002 10601022 PMC1171763

[85] Nakagawa K Taya Y Tamai K Yamaizumi M. Requirement of ATM phosphorylation of the human p53 protein at serine 15 following DNA double-strand breaks. Mol Cell Biol (1999) 19(4):2828–34. 10082548 10.1128/mcb.19.4.2828 PMC84075

[86] Michael D Oren M. The p53-Mdm2 module and the ubiquitin system. Semin Cancer Biol (2003) 13(1):49–58. 10.1016/S1044-579X(02)00099-8 12507556

[87] Montes de Oca Luna R Wagner DS Lozano G Rescue of early embryonic lethality in mdm2-deficient mice by deletion of p53. Nature (1995) 378(6553):203–6 10.1038/378203a0 7477326

[88] Jones SN Roe AE Donehower LA Bradley A Rescue of embryonic lethality in Mdm2-deficient mice by absence of p53. Nature (1995) 378(6553):206–8 10.1038/378206a0 7477327

[89] Li M Brooks CL Wu-Baer F Chen D Baer R Gu W. Mono- versus polyubiquitination: differential control of p53 fate by Mdm2. Science (2003) 302(5652):1972–5. 10.1126/science.1091362 14671306

[90] Yu GW Rudiger S Veprintsev D Freund S Fernandez-Fernandez MR Fersht AR. The central region of HDM2 provides a second binding site for p53. Proc Natl Acad Sci U S A (2006) 103(5):1227–32. 10.1073/pnas.0510343103 16432196 PMC1360574

[91] Xirodimas DP Saville MK Bourdon JC Hay RT Lane DP. Mdm2-mediated NEDD8 conjugation of p53 inhibits its transcriptional activity. Cell (2004) 118(1):83–97. 10.1016/j.cell.2004.06.016 15242646

[92] Oliner JD Kinzler KW Meltzer PS George DL Vogelstein B. Amplification of a gene encoding a p53-associated protein in human sarcomas. Nature (1992) 358(6381):80–3. 10.1038/358080a0 1614537

[93] Candau R Scolnick DM Darpino P Ying CY Halazonetis TD Berger SL. Two tandem and independent sub-activation domains in the amino terminus of p53 require the adaptor complex for activity. Oncogene (1997) 15(7):807–16. 10.1038/sj.onc.1201244 9266967

[94] Vogelstein B Lane D Levine AJ Surfing the p53 network. Nature (2000) 408(6810):307–10 10.1038/35042675 11099028

[95] Appella E Anderson CW. Post-translational modifications and activation of p53 by genotoxic stresses. Eur J Biochem (2001) 268(10):2764–72. 10.1046/j.1432-1327.2001.02225.x 11358490

[96] Saito S Yamaguchi H Higashimoto Y Chao C Xu Y Fornace AJ Jr Phosphorylation site interdependence of human p53 post-translational modifications in response to stress. J Biol Chem (2003) 278(39):37536–44. 10.1074/jbc.M305135200 12860987

[97] Shvarts A Steegenga WT Riteco N Van Laar T Dekker P Bazuine M MDMX: a novel p53-binding protein with some functional properties of MDM2. EMBO J (1996) 15(19):5349–57. 8895579 PMC452278

[98] Shvarts A Bazuine M Dekker P Ramos YF Steegenga WT Merckx G Isolation and identification of the human homolog of a new p53-binding protein, Mdmx. Genomics (1997) 43(1):34–42. 10.1006/geno.1997.4775 9226370

[99] Böttger V Böttger A Garcia-Echeverria C Ramos YF van der Eb AJ Jochemsen AG Comparative study of the p53-mdm2 and p53-MDMX interfaces. Oncogene (1999) 18(1):189–99. 10.1038/sj.onc.1202281 9926934

[100] Tanimura S Ohtsuka S Mitsui K Shirouzu K Yoshimura A Ohtsubo M. MDM2 interacts with MDMX through their RING finger domains. FEBS Lett (1999) 447(1):5–9. 10.1016/S0014-5793(99)00254-9 10218570

[101] Sharp DA Kratowicz SA Sank MJ George DL. Stabilization of the MDM2 oncoprotein by interaction with the structurally related MDMX protein. J Biol Chem (1999) 274(53):38189–96. 10.1074/jbc.274.53.38189 10608892

[102] Danovi D Meulmeester E Pasini D Migliorini D Capra M Frenk R Amplification of MdmX (or Mdm4) directly contributes to tumor formation by inhibiting p53 tumor. Mol Cell Biol (2004) 24(13):5835–43. 10.1128/MCB.24.13.5835-5843.2004 15199139 PMC480894

[103] Ramos YF Stad R Attema J Peltenburg LT van der Eb AJ Jochemsen AG. Aberrant expression of HDMX proteins in tumor cells correlates with wild-type p53. Cancer Res (2001) 61(5):1839–42. 11280734

[104] Sabbatini P McCormick F. MDMX inhibits the p300/CBP-mediated acetylation of p53. DNA Cell Biol (2002) 21(7):519–25. 10.1089/104454902320219077 12162806

[105] Brooks CL Gu W. The impact of acetylation and deacetylation on the p53 pathway. Protein Cell (2011) 2(6):456–62. 10.1007/s13238-011-1063-9 21748595 PMC3690542

[106] Walker KK Levine AJ. Identification of a novel p53 functional domain that is necessary for efficient growth suppression. Proc Natl Acad Sci U S A (1996) 93(26):15335–40. 10.1073/pnas.93.26.15335 8986812 PMC26405

[107] Sakamuro D Sabbatini P White E Prendergast GC. The polyproline region of p53 is required to activate apoptosis but not growth arrest. Oncogene (1997) 15(8):887–98. 10.1038/sj.onc.1201263 9285684

[108] Zhu J Jiang J Zhou W Zhu K Chen X. Differential regulation of cellular target genes by p53 devoid of the PXXP motifs with impaired apoptotic activity. Oncogene (1999) 18(12):2149–55. 10.1038/sj.onc.1202533 10321740

[109] Berger M Vogt Sionov R Levine AJ Haupt Y. A role for the polyproline domain of p53 in its regulation by Mdm2. J Biol Chem (2001) 276(6):3785–90. 10.1074/jbc.M008879200 11053443

[110] Berger M Stahl N Del Sal G Haupt Y. Mutations in proline 82 of p53 impair its activation by Pin1 and Chk2 in response to DNA damage. Mol Cell Biol (2005) 25(13):5380–8. 10.1128/MCB.25.13.5380-5388.2005 15964795 PMC1156984

[111] Taylor JA Li Y He M Mason T Mettlin C Vogler WJ p53 mutations in bladder tumors from arylamine-exposed workers. Cancer Res (1996) 56(2):294–8. 8542583

[112] Sun XF Johannsson O Håkansson S Sellberg G Nordenskjöld B Olsson H A novel p53 germline alteration identified in a late onset breast cancer kindred. Oncogene (1996) 13(2):407–11. 8710380

[113] Bennett M Macdonald K Chan SW Luzio JP Simari R Weissberg P. Cell surface trafficking of Fas: a rapid mechanism of p53-mediated apoptosis. Science (1998) 282(5387):290–3. 10.1126/science.282.5387.290 9765154

[114] Gu W Roeder RG. Activation of p53 sequence-specific DNA binding by acetylation of the p53 C-terminal region. Cell (1997) 90(4):595–606. 10.1016/S0092-8674(00)80521-8 9288740

[115] Espinosa JM Emerson BM. Transcriptional regulation by p53 through intrinsic DNA/chromatin binding and site-directed cofactor recruitment. Mol Cell (2001) 8(1):57–69. 10.1016/S1097-2765(01)00283-0 11511360

[116] Dornan D Hupp TR. Inhibition of p53-dependent transcription by BOX-I phospho-peptide mimetics that bind to p300. EMBO Rep (2001) 2(2):139–44. 10.1093/embo-reports/kve025 11258706 PMC1083821

[117] Dornan D Shimizu H Perkins ND Hupp TR. DNA-dependent acetylation of p53 by the transcription coactivator p300. J Biol Chem (2003) 278(15):13431–41. 10.1074/jbc.M211460200 12499368

[118] Dornan D Shimizu H Burch L Smith AJ Hupp TR. The proline repeat domain of p53 binds directly to the transcriptional coactivator p300 and allosterically controls DNA-dependent acetylation of p53. Mol Cell Biol (2003) 23(23):8846–61. 10.1128/MCB.23.23.8846-8861.2003 14612423 PMC262654

[119] Ito A Lai CH Zhao X Saito S Hamilton MH Appella E P300/CBP-mediated p53 acetylation is commonly induced by p53-activating agents and inhibited by MDM2. EMBO J (2001) 20(6):1331–40. 10.1093/emboj/20.6.1331 11250899 PMC145533

[120] Liu L Scolnick DM Trievel RC Zhang HB Marmorstein R Halazonetis TD p53 sites acetylated in vitro by PCAF and p300 are acetylated in vivo in response to DNA damage. Mol Cell Biol (1999) 19(2):1202–9. 9891054 10.1128/mcb.19.2.1202 PMC116049

[121] Sakaguchi K Herrera JE Saito S Miki T Bustin M Vassilev A DNA damage activates p53 through a phosphorylation-acetylation cascade. Genes Dev (1998) 12(18):2831–41. 10.1101/gad.12.18.2831 9744860 PMC317174

[122] Knights CD Catania J Di Giovanni S Muratoglu S Perez R Swartzbeck A Distinct p53 acetylation cassettes differentially influence gene-expression patterns and cell fate. J Cell Biol (2006) 173(4):533–44. 10.1083/jcb.200512059 16717128 PMC2063863

[123] Wang J Qian J Hu Y Kong X Chen H Shi Q ArhGAP30 promotes p53 acetylation and function in colorectal cancer. Nat Commun (2014) 5:4735. 10.1038/ncomms5735 25156493

[124] Wang Y Reed M Wang P Stenger JE Mayr G Anderson ME P53 domains: identification and characterization of two autonomous DNA-binding regions. Genes Dev (1993) 7(12B):2575–86. 10.1101/gad.7.12b.2575 8276240

[125] Wu L Bayle JH Elenbaas B Pavletich NP Levine AJ. Alternatively spliced forms in the carboxy-terminal domain of the p53 protein regulate its ability to promote anneling of complementary single strands of nucleic acid. Mol Cell Biol (1995) 15(1):497–504. 7528329 10.1128/mcb.15.1.497 PMC231999

[126] Jayaraman L Prives C. Covalent and noncovalent modifiers of the p53 protein. Cell Mol Life Sci (1999) 55(1):76–87. 10.1007/s000180050271 10065153 PMC11146848

[127] Hupp TR Meek DW Midgley CA Lane DP Regulation of the specific DNA binding function of p53. Cell (1992) 71(5):875–86 10.1016/0092-8674(92)90562-Q 1423635

[128] Ahn J Prives C The C-terminus of p53: the more you learn the less you know. Nat Struct Biol (2001) 8(9):730–2 10.1038/nsb0901-730 11524665

[129] Friedler A Veprintsev DB Freund SM von Glos KI Fersht AR. Modulation of binding of DNA to the C-terminal domain of p53 by acetylation. Structure (2005) 13(4):629–36. 10.1016/j.str.2005.01.020 15837201

[130] Tafvizi A Huang F Leith JS Fersht AR Mirny LA van Oijen AM. Tumor suppressor p53 slides on DNA with low friction and high stability. Biophys J (2008) 95(1):L01–3. 10.1529/biophysj.108.134122 18424488 PMC2426630

[131] Tafvizi A Huang F Fersht AR Mirny LA van Oijen AM. A single-molecule characterization of p53 search on DNA. Proc Natl Acad Sci U S A (2011) 108(2):563–8. 10.1073/pnas.1016020107 21178072 PMC3021058

[132] Melero R Rajagopalan S Lázaro M Joerger AC Brandt T Veprintsev DB Electron microscopy studies on the quaternary structure of p53 reveal different binding modes for p53 tetramers in complex with DNA. Proc Natl Acad Sci U S A (2011) 108(2):557–62. 10.1073/pnas.1015520107 21178074 PMC3021029

[133] Juan LJ Shia WJ Chen MH Yang WM Seto E Lin YS Histone deacetylases specifically down-regulate p53-dependent gene activation. J Biol Chem (2000) 275(27):20436–43. 10.1074/jbc.M000202200 10777477

[134] Lee CW Wong LL Tse EY Liu HF Leong VY Lee JM AMPK promotes p53 acetylation via phosphorylation and inactivation of SIRT1 in liver cancer cells. Cancer Res (2012) 72(17):4394–404. 10.1158/0008-5472.CAN-12-0429 22728651 PMC3433393

[135] Lee SM Bae JH Kim MJ Lee HS Lee MK Chung BS Bcr-Abl-independent imatinib-resistant K562 cells show aberrant protein acetylation and increased sensitivity to histone deacetylase inhibitors. J Pharmacol Exp Ther (2007) 322(3):1084–92. 10.1124/jpet.107.124461 17569822

[136] Bots M Verbrugge I Martin BP Salmon JM Ghisi M Baker A Differentiation therapy for the treatment of t(8;21) acute myeloid leukemia using histone deacetylase inhibitors. Blood (2014) 123(9):1341–52. 10.1182/blood-2013-03-488114 24415537 PMC3938147

[137] Stratton MR Campbell PJ Futreal PA The cancer genome. Nature (2009) 458(7239):719–24 10.1038/nature07943 19360079 PMC2821689

[138] Weigelt B Geyer FC Reis-Filho JS. Histological types of breast cancer: how special are they? Mol Oncol (2010) 4(3):192–208. 10.1016/j.molonc.2010.04.004 20452298 PMC5527938

[139] Curtis C Shah SP Chin SF Turashvili G Rueda OM Dunning MJ The genomic and transcriptomic architecture of 2,000 breast tumors reveals novel subgroups. Nature (2012) 486(7403):346–52. 10.1038/nature10983 22522925 PMC3440846

[140] Schlomm T Iwers L Kirstein P Jessen B Köllermann J Minner S Clinical significance of p53 alterations in surgically treated prostate cancers. Mod Pathol (2008) 21(11):1371–8. 10.1038/modpathol.2008.104 18552821

[141] Calabretta B Perrotti D The biology of CML blast crisis. Blood (2004) 103(11):4010–22 10.1182/blood-2003-12-4111 14982876

[142] Malcikova J Pavlova S Kozubik KS Pospisilova S. TP53 mutation analysis in clinical practice: lessons from chronic lymphocytic leukemia. Hum Mutat (2014) 35(6):663–71. 10.1002/humu.22508 24415659

[143] Scheffner M Werness BA Huibregtse JM Levine AJ Howley PM. The E6 oncoprotein encoded by human papillomavirus types 16 and 18 promotes the degradation of p53. Cell (1990) 63(6):1129–36. 10.1016/0092-8674(90)90409-8 2175676

[144] Terrier O Bourdon JC Rosa-Calatrava M p53 protein isoforms: key regulators in the front line of pathogen infection? PLoS Pathog (2013) 9(4):e1003246 10.1371/journal.ppat.1003246 23592981 PMC3616980

[145] Aguilar F Hussain SP Cerutti P. Aflatoxin B1 induces the transversion of G–T in codon 249 of the p53 tumor suppressor gene in human hepatocytes. Proc Natl Acad Sci U S A (1993) 90(18):8586–90. 10.1073/pnas.90.18.8586 8397412 PMC47402

[146] Leroy B Girard L Hollestelle A Minna JD Gazdar AF Soussi T. Analysis of TP53 mutation status in human cancer cell lines: a reassessment. Hum Mutat (2014) 35(6):756–65. 10.1002/humu.22556 24700732 PMC4451114

[147] Masuda H Miller C Koeffler HP Battifora H Cline MJ. Rearrangement of the p53 gene in human osteogenic sarcomas. Proc Natl Acad Sci U S A (1987) 84(21):7716–9. 10.1073/pnas.84.21.7716 2823272 PMC299371

[148] Barretina J Taylor BS Banerji S Ramos AH Lagos-Quintana M Decarolis PL Subtype-specific genomic alterations define new targets for soft-tissue sarcoma therapy. Nat Genet (2010) 42(8):715–21. 10.1038/ng.619 20601955 PMC2911503

[149] Li FP Fraumeni JF Jr Rhabdomyosarcoma in children: epidemiologic study and identification of a familial cancer syndrome. J Natl Cancer Inst (1969) 43(6):1365–73. 5396222

[150] Li FP Fraumeni F Jr Soft-tissue sarcomas, breast cancer, and other neoplasms. A familiar syndrome? Ann Intern Med (1969) 71(4):747–52 10.7326/0003-4819-71-4-747 5360287

[151] Malkin D Li FP Strong LC Fraumeni JF Jr Nelson CE Kim DH Germ line p53 mutations in a familiar syndrome of breast cancer, sarcomas, and other neoplasms. Science (1990) 250(4985):1233–8. 10.1126/science.1978757 1978757

[152] Marutani M Tonoki H Tada M Takahashi M Kashiwazaki H Hida Y. Dominant-negative mutations of the tumor suppressor p53 relating to early onset of glioblastoma multiforme. Cancer Res (1999) 59(19):4765–9. 10519380

[153] Kato S Han SY Liu W Otsuka K Shibata H Kanamaru R Understanding the function-structure and function-mutation relationships of the p53 tumor suppressor protein by high-resolution missense mutation analysis. Proc Natl Acad Sci U S A (2003) 100(14):8424–9. 10.1073/pnas.1431692100 12826609 PMC166245

[154] Kenzelmann Broz D Attardi LD. In vivo analysis of p53 tumor suppressor function using genetically engineered mouse models. Carcinogenesis (2010) 31(8):1311–8. 10.1093/carcin/bgp331 20097732 PMC2915627

[155] Christophorou MA Ringshausen I Finch AJ Swigart LB Evan GI. The pathological response to DNA damage does not contribute to p53-mediated tumor suppression. Nature (2006) 443(7108):214–7. 10.1038/nature05077 16957739

[156] Efeyan A Collado M Velasco-Miguel S Serrano M. Genetic dissection of the role of p21Cip1/Waf1 in p53-mediated tumor suppression. Oncogene (2007) 26(11):1645–9. 10.1038/sj.onc.1209972 16964282

[157] Velente LJ Gray DH Michalak EM Pinon-Hofbauer J Egle A Scott CL p53 efficiently suppresses tumor development in the complete absence of its cell-cycle inhibitory and proapoptotic effectors p21, Puma and Noxa. Cell Rep (2013) 3(5):1339–45. 10.1016/j.celrep.2013.04.012 23665218

[158] Li T Kon N Jiang L Tan M Ludwig T Zhao Y Tumor suppression in the absence of p53-mediated cell-cycle arrest, apoptosis and senescence. Cell (2012) 149(6):1269–83. 10.1016/j.cell.2012.04.026 22682249 PMC3688046

[159] Brady CA Jiang D Mello SS Johnson TM Jarvis LA Kozak MM Distinct p53 transcriptional programs dictate acute DNA-damage responses and tumor suppression. Cell (2011) 145(4):571–83. 10.1016/j.cell.2011.03.035 21565614 PMC3259909

[160] Wolf D Harris N Rotter V. Reconstitution of p53 expression in a nonproducer Ab-MuLV-transformed cell line by transfection of a functional p53 gene. Cell (1984) 38(1):119–26. 10.1016/0092-8674(84)90532-4 6088057

[161] Halevy O Michalovitz D Oren M. Different tumor-derived p53 mutants exhibit distinct biological activities. Science (1990) 250(4977):113–6. 10.1126/science.2218501 2218501

[162] Shaulsky G Goldfinger N Rotter V. Alterations in tumor development in vivo mediated by expression of wild type or mutant p53 proteins. Cancer Res (1991) 51(19):5232–7. 1717142

[163] Milner J Medcalf EA Cook AC. Tumor suppressor p53: analysis of wild-type and mutant p53 complexes. Mol Cell Biol (1991) 11(1):12–9. 1986215 10.1128/mcb.11.1.12-19.1991 PMC359578

[164] Milner J Medcalf EA. Cotranslation of activated mutant p53 with wild type drives the wild-type p53 protein into the mutant conformation. Cell (1991) 65(5):765–74. 10.1016/0092-8674(91)90384-B 2040013

[165] Joerger AC Rajagopalan S Natan E Veprintsev DB Robinson CV Fersht AR. Structural evolution of p53, p63, and p73: implication for heterotetramer formation. Proc Natl Acad Sci U S A (2009) 106(42):17705–10. 10.1073/pnas.0905867106 19815500 PMC2764906

[166] Xu J Reumers J Couceiro JR De Smet F Gallardo R Rudyak S Gain of function of mutant p53 by coaggregation with multiple tumor suppressors. Nat Chem Biol (2011) 7(5):285–95. 10.1038/nchembio.546 21445056

[167] Muller PA Vousden KH p53 mutations in cancer. Nat Cell Biol (2013) 15(1):2–8 10.1038/ncb2641 23263379

[168] Bisio A Ciribilli Y Fronza G Inga A Monti P. TP53 mutants in the tower of babel of cancer progression. Hum Mutat (2014) 35(6):689–701. 10.1002/humu.22514 24449472

[169] Fan PD Cong F Goff SP. Homo- and hetero-oligomerization of the c-Abl kinase and Abelson-interactor-1. Cancer Res (2003) 63(4):873–7. 12591740

[170] McWhirter JR Galasso DL Wang JY. A coiled-coil oligomerization domain of Bcr is essential for the transforming function of Bcr-Abl oncoproteins. Mol Cell Biol (1993) 13(12):7587–95. 8246975 10.1128/mcb.13.12.7587-7595.1993 PMC364830

[171] Chemes LB Noval MG Sánchez IE de Prat-Gay G. Folding of a cyclin box linking multitarget binding to marginal stability, oligomerization, and aggregation of the retinoblastoma tumor suppressor AB pocket domain. J Biol Chem (2013) 288(26):18923–38. 10.1074/jbc.M113.467316 23632018 PMC3696668

[172] Lee M Sadowska A Bekere I Ho D Gully BS Lu Y The structure of human SFPQ reveals a coiled-coil mediated polymer essential for functional aggregation in gene regulation. Nucleic Acids Res (2015) pii:gkv156. 10.1093/nar/gkv156 25765647 PMC4402515

[173] Bullock AN Henckel J DeDecker BS Johnson CM Nikolova PV Proctor MR Thermodynamic stability of wild-type and mutant p53 core domain. Proc Natl Acad Sci U S A (1997) 94(26):14338–42. 10.1073/pnas.94.26.14338 9405613 PMC24967

[174] Ishimaru D Andrade LR Taixeira LS Quesado PA Maiolino LM Lopez PM Fibrillar aggregates of the tumor suppressor p53 core domain. Biochemistry (2003) 42(30):9022–7. 10.1021/bi034218k 12885235

[175] Ishimaru D Maia LF Maiolino LM Quesado PA Lopez PC Almeida FC Conversion of wild-type p53 core domain into a conformation that mimics a hot-spot mutant. J Mol Biol (2003) 333(2):443–51. 10.1016/j.jmb.2003.08.026 14529628

[176] Ishimaru D Lima LM Maia LF Lopez PM Ano Bom AP Valente AP Reversible aggregation plays a crucial role on the folding landscape of p53 core domain. Biophys J (2004) 87(4):2691–700. 10.1529/biophysj.104.044685 15298872 PMC1304688

[177] Wang G Fersht AR. Propagation of aggregated p53: cross-reaction and coaggregation vs. seeding. Proc Natl Acad Sci U S A (2015) 112(8):2443–8. 10.1073/pnas.1500262112 25675527 PMC4345553

[178] Wang G Fersht AR. Mechanism of initiation of aggregation of p53 revealed by ϕ-value analysis. Proc Natl Acad Sci U S A (2015) 112(8):2437–42. 10.1073/pnas.1500243112 25675526 PMC4345617

[179] Lasagna-Reeves CA Clos AL Castillo-Carranza D Sengupta U Guerrero-Muñoz M Kelly B Dual role of p53 amyloid formation in cancer; loss of function and gain of toxicity. Biochem Biophys Res Commun (2013) 430(3):963–8. 10.1016/j.bbrc.2012.11.130 23261448

[180] Kluth M Harasimowicz S Burkhardt L Grupp K Krohn A Prien K Clinical significance of different types of p53 gene alteration in surgically treated prostate cancer. Int J Cancer (2014) 135(6):1369–80. 10.1002/ijc.28784 24523142

[181] Yang-Hartwich Y Soteras MG Lin ZP Holmberg J Sumi N Craveiro V p53 protein aggregation promotes platinum resistance in ovarian cancer. Oncogene (2014). 10.1038/onc.2014.296 25263447

[182] Silva JL De Moura Gallo CV Costa DC Rangel LP Prion-like aggregation of mutant p53 in cancer. Trends Biochem Sci (2014) 39(6):260–7 10.1016/j.tibs.2014.04.001 24775734

[183] Prusiner SB. Prions. Proc Natl Acad Sci U S A (1998) 95(23):13363–83. 10.1073/pnas.95.23.13363 9811807 PMC33918

[184] Prusiner SB Scott MR DeArmond SJ Cohen FE Prion protein biology. Cell (1998) 93(3):337–48 10.1016/S0092-8674(00)81163-0 9590169

[185] Cordeiro Y Machado F Juliano L Juliano MA Brentani RR Foguel D DNA converts cellular prion protein into the *β*-sheet conformation and inhibits prion peptide aggregation. J Biol Chem (2001) 276(52):49400–9. 10.1074/jbc.M106707200 11604397

[186] Silva JL Lima LM Foguel D Cordeiro Y. Intriguing nucleic-acid-binding features of mammalian prion protein. Trends Biochem Sci (2008) 33(3):132–40. 10.1016/j.tibs.2007.11.003 18243708

[187] Gomes MP Millen TA Ferreira PS Silva NL Vieira TC Almeida MS Prion protein complexed to N2a cellular RNAs through its N-terminal domain forms aggregates and is toxic to murine neuroblastoma cells. J Biol Chem (2008) 283:19616–25. 10.1074/jbc.M802102200 18456654 PMC2443653

[188] Lee SH Lee SJ Chung JY Jung YS Choi SY Hwang SH p53 secreted by K-Ras-Snail pathway, is endocytosed by K-Ras-mutated cells; implication of target-specific drug delivery and early diagnostic marker. Oncogene (2009) 28(19):2005–14. 10.1038/onc.2009.67 19347028

[189] Lee SH Woo TG Lee SJ Kim JS Ha NC Park BJ. Extracellular p53 fragment re-enters K-Ras mutated cells through the caveolin-1 dependent early endosomal system. Oncotarget (2013) 4(12):2523–31. 24344114 10.18632/oncotarget.1550 PMC3926846

[190] Forget KJ Tremblay G Roucou X. p53 aggregates penetrate cells and induce the co-aggregation of the intracellular p53. PLoS One (2013) 8(7):e69242. 10.1371/journal.pone.0069242 23844254 PMC3700952

[191] Halaschek-Wiener J Wacheck V Kloog Y Jansen B. Ras inhibition leads to transcriptional activation of p53 and down-regulation of Mdm2: two mechanisms that cooperatively increase p53 function in colon cancer cells. Cell Signal (2004) 16(11):1319–27. 10.1016/j.cellsig.2004.04.003 15337531

[192] Lee SH Lee SJ Jung YS Xu Y Kang HS Ha NC Blocking of p53-Snail binding, promoted by oncogenic K-Ras, recovers p53 expression and function. Neoplasia (2009) 11(1):22–31. 19107228 10.1593/neo.81006 PMC2606115

[193] Karnoub AE Weinberg RA. Ras oncogenes: split personalities. Nat Rev Mol Cell Biol (2008) 9(7):517–31. 10.1038/nrm2438 18568040 PMC3915522

[194] Friedler A Veprintsev DB Hansson LO Fersht AR. Kinetic instability of p53 core domain mutants: implications for rescue by small molecules. J Biol Chem (2003) 278(26):24108–12. 10.1074/jbc.M302458200 12700230

[195] Lambert JM Gorzov P Veprintsev DB Söderqvist M Segerbäck D Bergman J PRIMA-1 reactivates mutant p53 by covalent binding to the core domain. Cancer Cell (2009) 15(5):376–88. 10.1016/j.ccr.2009.03.003 19411067

[196] Ferraz da Costa DC Casanova FA Quarti J Malheiros MS Sanches D Dos Santos PS Transient transfection of a wild-type p53 gene triggers resveratrol-induced apoptosis in cancer cells. PLoS One (2012) 7(11):e48746. 10.1371/journal.pone.0048746 23152798 PMC3495968

[197] Cowan-Jacob SW. Structural biology of protein tyrosine kinases. Cell Mol Life Sci (2006) 63(22):2608–25. 10.1007/s00018-006-6202-8 17041812 PMC11136174

[198] Franz WM Berger P Wang JY. Deletion of an N-terminal regulatory domain of the c-abl tyrosine kinase activates its oncogenic potential. EMBO J (1989) 8(1):137–47. 2496972 10.1002/j.1460-2075.1989.tb03358.x PMC400782

[199] Jackson P Baltimore D. N-terminal mutations activate the leukemogenic potential of the myristoylated form of c-abl. EMBO J (1989) 8(2):449–56. 2542016 10.1002/j.1460-2075.1989.tb03397.x PMC400826

[200] Mayer BJ Baltimore D. Mutagenic analysis of the roles of SH2 and SH3 domains in regulation of the Abl tyrosine kinase. Mol Cell Biol (1994) 14(5):2883–94. 8164650 10.1128/mcb.14.5.2883-2894.1994 PMC358656

[201] Chen S O’Reilly LP Smithgall TE Engen JR. Tyrosine phosphorylation in the SH3 domain disrupts negative regulatory interactions within the c-Abl kinase core. J Mol Biol (2008) 383(2):414–23. 10.1016/j.jmb.2008.08.040 18775435 PMC2596866

[202] Ren R Mayer BJ Cicchetti P Baltimore D. Identification of a ten-amino acid proline-rich SH3 binding site. Science (1993) 259(5098):1157–61. 10.1126/science.8438166 8438166

[203] Barilá D Superti-Furga G. An intramolecular SH3-domain interaction regulates c-Abl activity. Nat Genet (1998) 18(3):280–2. 10.1038/ng0398-280 9500553

[204] Chen S Brier S Smithgall TE Engen JR. The Abl SH2-kinase linker naturally adopts a conformation competent for SH3 domain binding. Protein Sci (2007) 16(4):572–81. 10.1110/ps.062631007 17327393 PMC2203333

[205] Pendergast AM Muller AJ Havlik MH Clark R McCormick F Witte ON. Evidence for regulation of the human ABL tyrosine kinase by a cellular inhibitor. Proc Natl Acad Sci U S A (1991) 88(13):5927–31. 10.1073/pnas.88.13.5927 1712111 PMC51991

[206] Welch PJ Wang JY. A C-terminal protein-binding domain in the retinoblastoma protein regulates nuclear c-Abl tyrosine kinase in the cell cycle. Cell (1993) 75(4):779–90. 10.1016/0092-8674(93)90497-E 8242749

[207] Shi Y Alin K Goff SP. Abl-interactor-1, a novel SH3 protein binding to the carboxy-terminal portion of the Abl protein suppresses v-abl transforming activity. Genes Dev (1995) 9(21):2583–97. 10.1101/gad.9.21.2583 7590237

[208] Dai Z Pendergast AM. Abi-2, a novel SH3-containing protein interacts with the c-Abl tyrosine kinase and modulates c-Abl transforming activity. Genes Dev (1995) 9(21):2569–82. 10.1101/gad.9.21.2569 7590236

[209] Zhu J Shore SK. c-Abl tyrosine kinase activity is regulated by association with a novel SH3-domain-binding protein. Mol Cell Biol (1996) 16(12):7054–82. 8943360 10.1128/mcb.16.12.7054 PMC231708

[210] Wen ST Van Etten RA. The PAG gene product, a stress-induced protein with antioxidant properties, is an Abl SH3-binding protein and a physiological inhibitor of c-Abl tyrosine kinase activity. Genes Dev (1997) 11(19):2456–67. 10.1101/gad.11.19.2456 9334312 PMC316562

[211] Woodring PJ Hunter T Wang JY. Inhibition of c-Abl tyrosine kinase activity by filamentous actin. J Biol Chem (2001) 276(29):27104–10. 10.1074/jbc.M100559200 11309382

[212] Foray N Marot D Randrianarison V Venezia ND Picard D Perricaudet M Constitutive association of BRCA1 and c-Abl and its ATM-dependent disruption after irradiation. Mol Cell Biol (2002) 22(12):4020–32. 10.1128/MCB.22.12.4020-4032.2002 12024016 PMC133860

[213] Pluk H Dorey K Superti-Furga G Autoinhibition of c-Abl. Cell (2002) 108(2):247–59 10.1016/S0092-8674(02)00623-2 11832214

[214] Nagar B Hantschel O Young MA Scheffzek K Veach D Bornmann W Structural basis for the autoinhibition of c-Abl tyrosine kinase. Cell (2003) 112(6):859–71. 10.1016/S0092-8674(03)00194-6 12654251

[215] Hantschel O Nagar B Guettler S Kretzschmar J Dorey K Kuriyan J A myristoyl/phosphotyrosine switch regulates c-Abl. Cell (2003) 112(6):845–57. 10.1016/S0092-8674(03)00191-0 12654250

[216] Nagar B Hantschel O Seeliger M Davies JM Weis WI Superti-Furga G Organization of the SH3-SH2 unit in active and inactive forms of the c-Abl tyrosine kinase. Mol Cell (2006) 21(6):787–98. 10.1016/j.molcel.2006.01.035 16543148

[217] de Oliveira GA Pereira EG Ferretti GD Valente AP Cordeiro Y Silva JL. Intramolecular dynamics within the N-Cap-SH3-SH2 regulatory unit of the c-Abl tyrosine kinase reveal targeting to the cellular membrane. J Biol Chem (2013) 288(39):28331–45. 10.1074/jbc.M113.500926 23928308 PMC3784749

[218] Wang JY. Controlling Abl: auto-inhibition and co-inhibition? Nat Cell Biol (2004) 6(1):3–7. 10.1038/ncb0104-3 14704671

[219] Xu W Harrison SC Eck MJ. Three-dimensional structure of the tyrosine kinase c-Src. Nature (1997) 385(6617):595–602. 10.1038/385595a0 9024657

[220] Xu W Doshi A Lei M Eck MJ Harrison SC. Crystal structures of c-Src reveal features of its autoinhibitory mechanism. Mol Cell (1999) 3(5):629–38. 10.1016/S1097-2765(00)80356-1 10360179

[221] Schindler T Sicheri F Pico A Gazit A Levitzki A Kuriyan J. Crystal structure of Hck in complex with a Src family-selective tyrosine kinase inhibitor. Mol Cell (1999) 3(5):639–48. 10.1016/S1097-2765(00)80357-3 10360180

[222] Cowan-Jacob SW Fendrich G Manley PW Jahnke W Fabbro D Liebetanz J The crystal structure of a c-Src complex in an active conformation suggests possible steps in c-Src activation. Structure (2005) 13(6):861–71. 10.1016/j.str.2005.03.012 15939018

[223] Patwardhan P Resh MD. Myristoylation and membrane binding regulate c-Src stability and kinase activity. Mol Cell Biol (2010) 30(17):4094–107. 10.1128/MCB.00246-10 20584982 PMC2937550

[224] Plattner R Irvin BJ Guo S Blackburn K Kazlauskas A Abraham RT A new link between the c-Abl tyrosine kinase and phosphoinositide signaling through PLC-gamma1. Nat Cell Biol (2003) 5(4):309–19. 10.1038/ncb949 12652307

[225] Silverman L Resh MD. Lysine residues form an integral component of a novel NH2-terminal membrane targeting motif for myristylated pp60v-src. J Cell Biol (1992) 119(2):415–25. 10.1083/jcb.119.2.415 1400583 PMC2289653

[226] Resh MD Myristylation and palmitylation of Src family members: the fats of the matter. Cell (1994) 76(3):411–3 10.1016/0092-8674(94)90104-X 8313462

[227] Huse M Kuriyan J The conformational plasticity of protein kinases. Cell (2002) 109(3):275–82 10.1016/S0092-8674(02)00741-9 12015977

[228] Grebien F Hantschel O Wojcik J Kaupe I Kovacic B Wyrzucki AM Targeting the SH2-kinase interface in Bcr-Abl inhibits leukemogenesis. Cell (2011) 147(2):306–19. 10.1016/j.cell.2011.08.046 22000011 PMC3202669

[229] Zhou HX. How often does the myristoylated N-terminal latch of c-Abl come off? FEBS Lett (2003) 552(2–3):160–2. 10.1016/S0014-5793(03)00911-6 14527680

[230] Brasher BB Van Etten RA. c-Abl has high intrinsic tyrosine activity that is stimulated by mutation of the Src homology 3 domain and by autophosphorylation at two distinct regulatory tyrosines. J Biol Chem (2000) 275(45):35631–7. 10.1074/jbc.M005401200 10964922

[231] Frasca F Vigneri P Vella V Vigneri R Wang JY. Tyrosine kinase inhibitor STI571 enhances thyroid cancer cell motile response to hepatocyte growth factor. Oncogene (2001) 20(29):3845–56. 10.1038/sj.onc.1204531 11439348

[232] Plattner R Kadlec L DeMali KA Kazlauskas A Pendergast AM. c-Abl is activated by growth factors and Src family kinases and has a role in the cellular response to PDGF. Genes Dev (1999) 13(18):2400–11. 10.1101/gad.13.18.2400 10500097 PMC317022

[233] Kadlec L Pendergast AM. The amphiphysin-like protein 1 (ALP1) interacts functionally with the cAbl tyrosine kinase and may play a role in cytoskeletal regulation. Proc Natl Acad Sci U S A (1997) 94(23):12390–5. 10.1073/pnas.94.23.12390 9356459 PMC24959

[234] Tybulewicz VL Crawford CE Jackson PK Bronson RT Mulligan RC. Neonatal lethality and lymphopenia in mice with a homozygous disruption of the c-abl proto-oncogene. Cell (1991) 65(7):1153–63. 10.1016/0092-8674(91)90011-M 2065352

[235] Schwartzberg PL Stall AM Hardin JD Bowdish KS Humaran T Boast S Mice homozygous for the ablm1 mutation show poor viability and depletion of selected B and T cell populations. Cell (1991) 65(7):1165–75. 10.1016/0092-8674(91)90012-N 2065353

[236] Van Etten RA Jackson P Baltimore D. The mouse type IV c-abl gene product is a nuclear protein, and activation of transforming ability is associated with cytoplasmic localization. Cell (1989) 58(4):669–78. 10.1016/0092-8674(89)90102-5 2670246

[237] Wen ST Jackson PK Van Etten RA. The cytostatic function of c-Abl is controlled by multiple nuclear localization signals and requires the p53 and Rb tumor suppressor gene products. EMBO J (1996) 15(7):1583–95. 8612582 PMC450068

[238] Taagepera S McDonald D Loeb JE Whitaker LL McElroy AK Wang JY Nuclear-cytoplasmic shuttling of the c-Abl tyrosine kinase. Proc Natl Acad Sci U S A (1998) 95(13):7457–62. 10.1073/pnas.95.13.7457 9636171 PMC22649

[239] Kipreos ET Wang JY. Cell cycle-regulated binding of c-Abl tyrosine kinase to DNA. Science (1992) 256(5055):382–5. 10.1126/science.256.5055.382 1566087

[240] Hantschel O Wiesner S Güttler T Mackereth CD Rix LL Mikes Z Structural basis for the cytoskeletal association of Bcr-Abl/c-Abl. Mol Cell (2005) 19(4):461–73. 10.1016/j.molcel.2005.06.030 16109371

[241] Miao YJ Wang JY. Binding of A/T-rich DNA by three high mobility group-like domains in c-Abl tyrosine kinase. J Biol Chem (1996) 271(37):22823–30. 10.1074/jbc.271.37.22823 8798460

[242] David-Cordonnier MH Hamdane M Bailly C D’Halluin JC. Determination of the human c-Abl consensus DNA binding site. FEBS Lett (1998) 424(3):177–82. 10.1016/S0014-5793(98)00169-0 9539146

[243] Buchkovich K Duffy LA Harlow E. The retinoblastoma protein is phosphorylated during specific phases of the cell cycle. Cell (1989) 58(6):1097–105. 10.1016/0092-8674(89)90508-4 2673543

[244] Welch PJ Wang JY. Disruption of the retinoblastoma protein function by coexpression of its C pocket fragment. Genes Dev (1995) 9(1):31–46. 10.1101/gad.9.1.31 7828850

[245] Sawyers CL McLaughlin J Goga A Havlik M Witte O The nuclear tyrosine kinase c-Abl negatively regulates cell growth. Cell (1994) 77(1):121–31 10.1016/0092-8674(94)90240-2 7512450

[246] Goga A Liu X Hambuch TM Senechal K Major E Berk AJ p53 dependent growth suppression by the c-Abl nuclear tyrosine kinase. Oncogene (1995) 11(4):791–9. 7651743

[247] Kelly WG Dahmus ME Hart GW. RNA polymerase II is a glycoprotein. Modification of the COOH-terminal domain by O-GlcNac. J Biol Chem (1993) 268(14):10416–24. 8486697

[248] Baskaran R Dahmus ME Wang JY. Tyrosine phosphorylation of mammalian RNA polymerase II carboxy-terminal domain. Proc Natl Acad Sci U S A (1993) 90(23):11167–71. 10.1073/pnas.90.23.11167 7504297 PMC47943

[249] Duyster J Baskaran R Wang JY. Src homology 2 domain as a specificity determinant in the c-Abl-mediated tyrosine phosphorylation of the RNA polymerase II carboxy-terminal repeated domain. Proc Natl Acad Sci U S A (1995) 92(5):1555–9. 10.1073/pnas.92.5.1555 7533294 PMC42558

[250] Baskaran R Chiang GG Wang JY. Identification of a binding site in c-Abl1 tyrosine kinase for the C-terminal repeated domain of RNA polymerase II. Mol Cell Biol (1996) 16(7):3361–9. 8668151 10.1128/mcb.16.7.3361 PMC231330

[251] Baskaran R Wood LD Whitaker LL Canman CE Morgan SE Xu Y Ataxia telangiectasia mutant protein activates c-Abl tyrosine kinase in response to ionizing radiation. Nature (1997) 387(6632):516–9. 10.1038/387516a0 9168116

[252] Shafman T Khanna KK Kedar P Spring K Kozlov S Yen T Interaction between ATM protein and c-Abl in response to DNA damage. Nature (1997) 387(6632):520–3. 10.1038/387520a0 9168117

[253] Khosravi R Maya R Gottlieb T Oren M Shiloh Y Shkedy D. Rapid ATM-dependent phosphorylation of MDM2 precedes p53 accumulation in response to DNA damage. Proc Natl Acad Sci U S A (1999) 96(26):14973–7. 10.1073/pnas.96.26.14973 10611322 PMC24757

[254] Maya R Balass M Kim ST Shkedy D Leal JF Shifman O ATM-dependent phosphorylation of Mdm2 on serine 395: role in p53 activation by DNA damage. Genes Dev (2001) 15(9):1067–77. 10.1101/gad.886901 11331603 PMC312683

[255] Goldberg Z Vogt Sionov R Berger M Zwang Y Perets R Van Etten RA Tyrosine kinase phosphorylation of Mdm2 by c-Abl: implications for p53 regulation. EMBO J (2002) 21(14):3715–27. 10.1093/emboj/cdf384 12110584 PMC125401

[256] Sionov RV Moallem E Berger M Kazaz A Gerlitz O Ben-Neriah Y c-Abl neutralizes the inhibitory effect of Mdm2 on p53. J Biol Chem (1999) 274(13):8371–4. 10.1074/jbc.274.13.8371 10085066

[257] Sionov RV Coen S Goldberg Z Berger M Bercovich B Ben-Neriah Y c-Abl regulates p53 levels under normal and stress conditions by preventing its nuclear export and ubiquitination. Mol Cell Biol (2001) 21(17):5869–78. 10.1128/MCB.21.17.5869-5878.2001 11486026 PMC87306

[258] Yuan ZM Huang Y Fan MM Sawyers C Kharbanda S Kufe D. Genotoxic drugs induce interaction of the c-Abl tyrosine kinase and the tumor suppressor protein p53. J Biol Chem (1996) 271(43):26457–60. 10.1074/jbc.271.43.26457 8900110

[259] Liu ZG Baskaran R Lea-Chou ET Wood LD Chen Y Karin M Three distinct signalling responses by murine fibroblasts to genotoxic stress. Nature (1996) 384(6606):273–6. 10.1038/384273a0 8918879

[260] Erol A. Deciphering the intricate regulatory mechanisms for the cellular choice between cell repair, apoptosis or senescence in response to damaging signals. Cell Signal (2011) 23(7):1076–81. 10.1016/j.cellsig.2010.11.023 21144894

[261] Van Etten RA. c-Abl regulation: a tail of two lipids. Curr Biol (2003) 13(15):R608–10. 10.1016/S0960-9822(03)00528-1 12906815

[262] Plattner R Koleske AJ Kazlauskas A Pendergast AM. Bidirectional signaling links the Abelson kinases to the platelet-derived growth factor receptor. Mol Cell Biol (2004) 24(6):2573–83. 10.1128/MCB.24.6.2573-2583.2004 14993293 PMC355852

[263] Van Etten RA Jackson PK Baltimore D Sanders MC Matsudaira PT Janmey PA. The COOH terminus of the c-Abl tyrosine kinase contains distinct F- and G-actin binding domains with bundling activity. J Cell Biol (1994) 124(3):325–40. 10.1083/jcb.124.3.325 8294516 PMC2119935

[264] Wang Y Miller AL Mooseker MS Koleske AJ. The Abl-related gene (Arg) nonreceptor tyrosine kinase uses two F-actin-binding domains to bundle F-actin. Proc Natl Acad Sci U S A (2001) 98(26):14865–70. 10.1073/pnas.251249298 11752434 PMC64950

[265] Lewis JM Baskaran R Taagepera S Schwartz MA Wang JY. Integrin regulation of c-Abl tyrosine kinase activity and cytoplasmic-nuclear transport. Proc Natl Acad Sci U S A (1996) 93(26):15174–9. 10.1073/pnas.93.26.15174 8986783 PMC26376

[266] Srinivasan D Plattner R. Activation of Abl tyrosine kinases promotes invasion of aggressive breast cancer cells. Cancer Res (2006) 66(11):5648–55. 10.1158/0008-5472.CAN-06-0734 16740702

[267] Lin J Sun T Ji L Deng W Roth J Minna J Oncogenic activation of c-Abl in non-small cell lung cancer cells lacking FUS1 expression: inhibition of c-Abl by the tumor suppressor gene product Fus1. Oncogene (2007) 26(49):6989–96. 10.1038/sj.onc.1210500 17486070 PMC3457636

[268] Rikova K Guo A Zeng Q Possemato A Yu J Haack H Global survey of phosphotyrosine signaling identifies oncogenic kinases in lung cancer. Cell (2007) 131(6):1190–203. 10.1016/j.cell.2007.11.025 18083107

[269] Ganguly SS Fiore LS Sims JT Friend JW Srinivasan D Thacker MA c-Abl and Arg are activated in human primary melanomas, promote melanoma cell invasion via distinct pathways, and drive metastatic progression. Oncogene (2012) 31(14):1804–16. 10.1038/onc.2011.361 21892207 PMC3235241

[270] Lin J Arlinghaus R. Activated c-Abl tyrosine kinase in malignant solid tumors. Oncogene (2008) 27(32):4385–91. 10.1038/onc.2008.86 18391983

[271] Greuber EK Smith-Pearson P Wang J Pendergast AM Role of ABL family kinases in cancer: from leukaemia to solid tumours. Nat Rev Cancer (2013) 13(8):559–71 10.1038/nrc3563 23842646 PMC3935732

[272] Abelson HT Rabstein LS A new tumor inducing variant of Moloney leukemia virus. Proc Am Assoc Cancer Res (1969) 10:1.

[273] Abelson HT Rabstein LS Lymphosarcoma: virus-induced thymic-independent disease in mice. Cancer Res (1970) 30(8):2213–22. 4318922

[274] Nowell PC Hungerford DA A minute chromosome in human chronic granulocytic leukemia. Science (1960) 142:1497. 10.1126/science.144.3623.1229 14150328

[275] Rowley JD A new consistent chromosomal abnormality in chronic myelogenous leukaemia identified by quinacrine fluorescence and giemsa staining. Nature (1973) 243(5405):290–3 10.1038/243290a0 4126434

[276] Kurzrock R Kantarjian HM Druker BJ Talpaz M. Philadelphia chromosome-positive leukemias: from basic mechanisms to molecular therapeutics. Ann Intern Med (2003) 138(10):819–30. 10.7326/0003-4819-138-10-200305200-00010 12755554

[277] Jiang XY Trujillo JM Liang JC. Chromosomal breakpoints within the first intron of the ABL gene are nonrandom in patients with chronic myelogenous leukemia. Blood (1990) 76(3):597–601. 2198962

[278] Wetzler M Talpaz M Van Etten RA Hirsh-Ginsberg C Beran M Kurzrock R. Subcellular localization of Bcr, Abl, and Bcr-Abl proteins in normal and leukemic cells and correlation of expression with myeloid differentiation. J Clin Invest (1993) 92(4):1925–39. 10.1172/JCI116786 8408645 PMC288359

[279] Hoover RR Gerlach MJ Koh EY Daley GQ. Cooperative and redundant effects of STAT5 and Ras signaling in BCR/ABL transformed hematopoietic cells. Oncogene (2001) 20(41):5826–35. 10.1038/sj.onc.1204549 11593388

[280] Sánchez-García I Grütz G. Tumorigenic activity of the BCR-ABL oncogenes is mediated by BCL2. Proc Natl Acad Sci U S A (1995) 92(12):5287–91. 10.1073/pnas.92.12.5287 7777499 PMC41679

[281] Amarante-Mendes GP McGahon AJ Nishioka WK Afar DE Witte ON Green DR. Bcl-2-independent Bcr-Abl-mediated resistance to apoptosis: protection is correlated with up regulation of Bcl-xL. Oncogene (1998) 16(11):1383–90. 10.1038/sj.onc.1201664 9525737

[282] Neshat MS Raitano AB Wang HG Reed JC Sawyers CL. The survival function of the Bcr-Abl oncogene is mediated by Bad-dependent and -independent pathways: roles for phosphatidylinositol 3-kinase and raf. Mol Cell Biol (2000) 20(4):1179–86. 10.1128/MCB.20.4.1179-1186.2000 10648603 PMC85238

[283] Deming PB Schafer ZT Tashker JS Potts MB Deshmukh M Kornbluth S. Bcr-Abl-mediated protection from apoptosis downstream of mitochondrial cytochrome *c* release. Mol Cell Biol (2004) 24(23):10289–99. 10.1128/MCB.24.23.10289-10299.2004 15542838 PMC529043

[284] Druker BJ Talpaz M Resta DJ Peng B Buchdunger E Ford JM Efficacy and safety of a specific inhibitor of the BCR-ABL tyrosine kinase in chronic myeloid leukemia. N Engl J Med (2001) 344(14):1031–7. 10.1056/NEJM200104053441401 11287972

[285] Druker BJ Inhibition of the Bcr-Abl tyrosine kinase as a therapeutic strategy for CML. Oncogene (2002) 21(56):8541–6 10.1038/sj.onc.1206081 12476300

[286] Druker BJ Sawyers CL Kantarjian H Resta DJ Reese SF Ford JM Activity of a specific inhibitor of the BCR-ABL tyrosine kinase in the blast crisis of chronic myeloid leukemia and acute lymphoblastic leukemia with the Philadelphia chromosome. N Engl J Med (2001) 344(14):1038–42. 10.1056/NEJM200104053441401 11287973

[287] Gorre ME Mohammed M Ellwood K Hsu N Paquette R Rao PN. Clinical resistance to STI-571 cancer therapy caused by BCR-ABL gene mutation or amplification. Science (2001) 293(5531):876–80. 10.1126/science.1062538 11423618

[288] Barthe C Cony-Makhoul P Melo JV Mahon JR Roots of clinical resistance to STI-571 cancer therapy. Science (2001) 293(5538):2163 10.1126/science.293.5538.2163a 11567109

[289] Hochhaus A Kreil S Corbin AS La Rosée P Müller MC Lahaye T Molecular and chromosomal mechanisms of resistance to imatinib (STI571) therapy. Leukemia (2002) 16(11):2190–6. 10.1038/sj.leu.2402741 12399961

[290] Donato NJ Wu JY Stapley J Gallick G Lin H Arlinghaus R Bcr-Abl independence and LYN kinase overexpression in chronic myelogenous leukemia cells selected for resistance to STI571. Blood (2003) 101(2):690–8. 10.1182/blood.V101.2.690 12509383

[291] Hegedus T Orfi L Seprodi A Váradi A Sarkadi B Kéri G Interaction of tyrosine kinase inhibitors with the human multidrug transporter proteins, MDR1 and MRP1. Biochim Biophys Acta (2002) 1587(2–3):318–25. 10.1016/S0925-4439(02)00095-9 12084474

[292] Widmer N Colombo S Buclin T Decosterd LA Functional consequence of MDR1 expression on imatinib intracellular concentrations. Blood (2003) 102(3):1142 10.1182/blood-2003-03-0993 12869489

[293] Chung YJ Kim TM Kim DW Namkoong H Kim HK Ha SA Gene expression signatures associated with the resistance to imatinib. Leukemia (2006) 20(9):1542–50. 10.1038/sj.leu.2404310 16855633

[294] Graham SM Jørgensen HG Allan E Pearson C Alcorn MJ Richmond L Primitive, quiescent, Philadelphia-positive stem cells from patients with chronic myeloid leukemia are insensitive to STI571 in vitro. Blood (2002) 99(1):319–25. 10.1182/blood.V99.1.319 11756187

[295] Chu S Holtz M Gupta M Bhatia R. BCR/ABL kinase inhibition by imatinib mesylate enhances MAP kinase activity in chronic myelogenous leukemia CD34+ cells. Blood (2004) 103(8):3167–74. 10.1182/blood-2003-04-1271 15070699

[296] Kronenwett R Butterweck U Steidl U Kliszewski S Neumann F Bork S Distinct molecular phenotype of malignant CD34(+) hematopoietic stem and progenitor cells in chronic myelogenous leukemia. Oncogene (2005) 24(34):5313–24. 10.1038/sj.onc.1208596 15806158

[297] Branford S Rudzki Z Walsh S Grigg A Arthur C Taylor K High frequency of point mutations clustered within the adenosine triphosphate-binding region of BCR/ABL in patients with chronic myeloid leukemia or Ph-positive acute lymphoblastic leukemia who develop imatinib (STI571) resistance. Blood (2002) 99(9):3472–5. 10.1182/blood.V99.9.3472 11964322

[298] Branford S Rudzki Z Walsh S Parkinson I Grigg A Szer J Detection of BCR-ABL mutations in patients with CML treated with imatinib is virtually always accompanied by clinical resistance, and mutations in the ATP phosphate-binding loop (P-loop) are associated with a poor prognosis. Blood (2003) 102(1):276–83. 10.1182/blood-2002-09-2896 12623848

[299] Jabbour E Kantarjian H Jones D Talpaz M Bekele N O’Brien S Frequency and clinical significance of BCR-ABL mutations in patients with chronic myeloid leukemia treated with imatinib mesylate. Leukemia (2006) 20(10):1767–73. 10.1038/sj.leu.2404318 16855631

[300] Corbin AS La Rosée P Stoffregen EP Druker BJ Deininger MW. Several Bcr-Abl kinase domain mutants associated with imatinib mesylate resistance remain sensitive to imatinib. Blood (2003) 101(11):4611–4. 10.1182/blood-2002-12-3659 12576318

[301] Schindler T Bornmann W Pellicena P Miller WT Clarkson B Kuriyan J. Structural mechanism for STI-571 inhibition of Abelson tyrosine kinase. Science (2000) 289(5486):1938–42. 10.1126/science.289.5486.1938 10988075

[302] Nagar B Bornmann WG Pellicena P Schindler T Veach DR Miller WT Crystal structures of the kinase domain of c-Abl in complex with the small molecule inhibitors PD173955 and imatinib (STI-571). Cancer Res (2002) 62(15):4236–43. 12154025

[303] Zhou T Parillon L Li F Wang Y Keats J Lamore S Crystal structure of the T315I mutant of AbI kinase. Chem Biol Drug Des (2007) 70(3):171–81. 10.1111/j.1747-0285.2007.00556.x 17718712

[304] Griswold IJ MacPartlin M Bumm T Goss VL O’Hare T Lee KA. Kinase domain mutants of Bcr-Abl exhibit altered transformation potency, kinase activity, and substrate utilization, irrespective of sensitivity to imatinib. Mol Cell Biol (2006) 26(16):6082–93. 10.1128/MCB.02202-05 16880519 PMC1592813

[305] Skaggs BJ Gorre ME Ryvkin A Burgess MR Xie Y Han Y Phosphorylation of the ATP-binding loop directs oncogenicity of drug-resistant BCR-ABL mutants. Proc Natl Acad Sci U S A (2006) 103(51):19466–71. 10.1073/pnas.0609239103 17164333 PMC1698443

[306] Azam M Seeliger MA Gray NS Kuriyan J Daley GQ. Activation of tyrosine kinases by mutation of the gatekeeper threonine. Nat Struct Mol Biol (2008) 15(10):1109–18. 10.1038/nsmb.1486 18794843 PMC2575426

[307] Adrián FJ Ding Q Sim T Velentza A Sloan C Liu Y Allosteric inhibitors of Bcr-Abl-dependent cell proliferation. Nat Chem Biol (2006) 2(2):95–102. 10.1038/nchembio760 16415863

[308] Zhang J Adrián FJ Jahnke W Cowan-Jacob SW Li AG Iacob RE Targeting Bcr-Abl by combining allosteric with ATP-binding-site inhibitors. Nature (2010) 463(7280):501–6. 10.1038/nature08675 20072125 PMC2901986

[309] Mian AA Oancea C Zhao Z Ottmann OG Ruthardt M. Oligomerization inhibition, combined with allosteric inhibition, abrogates the transformation potential of T315I-positive BCR/ABL. Leukemia (2009) 23(12):2242–7. 10.1038/leu.2009.194 19798092

[310] Svergun DI Koch MH Timmins PA May RP Small Angle X-ray and Neutron Scattering from Solutions of Biological Macromolecules. New York, NY: Oxford University Press (2013). 358 p.

[311] Tidow H Melero R Mylonas E Freund SM Grossmann JG Carazo JM. Quaternary structures of tumor suppressor p53 and a specific p53 DNA complex. Proc Natl Acad Sci U S A (2007) 104(30):12324–9. 10.1073/pnas.0705069104 17620598 PMC1941468

[312] Wells M Tidow H Rutherford TJ Markwick P Jensen MR Mylonas E Structure of tumor suppressor p53 and its intrinsically disordered N-terminal transactivation domain. Proc Natl Acad Sci U S A (2008) 105(15):5762–7. 10.1073/pnas.0801353105 18391200 PMC2311362

